# ChatGPT4o's theranostic performance in the management of thoracolumbar spine fractures

**DOI:** 10.3389/fsurg.2025.1524396

**Published:** 2025-02-25

**Authors:** Xuehai Jia, Litai Ma, Yi Yang, Yi Deng, Changyong Shen, Kerui Zhang, Ya Li

**Affiliations:** ^1^ Department of Orthopedics, Orthopedic Research Institute, West China Hospital, Sichuan University, Chengdu, Sichuan, China; ^2^ The First Affiliated Hospital of Shihezi University, Shihezi, Xinjiang Uyghur, China

**Keywords:** ChatGPT4o, thoracolumbar spine fractures, theranostic performance, clinical practice, AI in medicine

## Introduction

ChatGPT, developed by OpenAI (https://chat.openai.com), is a publicly accessible tool that utilizes advanced machine learning algorithms to process and analyze extensive data, generating responses to user inquiries. On May 13, 2024, OpenAI launched the ChatGPT4o model, which, according to information on the OpenAI website, represents the latest, fastest, and most advanced version. This model supports a context length of up to 128k tokens (equivalent to the length of a long novel) and offers multimodal capabilities, including text and image inputs, as well as text, image, and audio outputs (https://help.openai.com). While numerous studies have explored ChatGPT's potential applications and challenges in the biomedical field (1, 2), limited research has been conducted on the specific capabilities of ChatGPT4o in the medical domain. A REVIEW article (3) published in Frontiers in Surgery mentions that ChatGPT lacks sufficient expertise and background understanding in specialized fields. However, the application of ChatGPT4o may have the potential to change this situation. To validate this model, we investigate the theranostic performance of ChatGPT4o in managing thoracolumbar spine fractures to assess its potential effectiveness and applications in clinical practice.

## Method

For our evaluation, we formulated 38 clinical questions based on the diagnostic, treatment, and management guidelines for thoracolumbar fractures established by the Congress of Neurological Surgeons (CNS) (4–14) and the Chinese Medical Association (CMA) (15). We input all 38 questions into ChatGPT-4o (OpenAI, accessed November 3, 2024) without providing additional context or guidelines. Each question was posed once, and the initial generated response was recorded. To minimize variability, no iterative refinement of prompts was performed. The responses were anonymized and compiled in Supplementary Material S1. Each response was subsequently reviewed by three independent spine surgery experts, who evaluated the responses according to both the established guidelines and their own clinical experience. Each expert used a five-point Likert scale to rate the responses: (1) indicating completely incorrect; (2) more incorrect than correct; (3) an equal mix of correct and incorrect; (4) more correct than incorrect; and (5) completely correct. The median score from the three experts was used as the final rating to minimize bias.

## Result

When ChatGPT4o was presented with “yes or no” questions, it typically responded with comprehensive diagnostic criteria and therapeutic principles rather than a simple “yes” or “no.” According to our results (Table 1), 0 responses (0%) received a score of 1, 1 response (2.63%) received a score of 2, 1 response (2.63%) scored a 3, 8 responses (21.05%) scored a 4, and 28 responses (73.68%) scored a 5. Approximately 94.7% of the responses were largely or entirely accurate.

**Table 1:** Five-point Likert scores for responses from inquires posed to chat-GPT4o.

Questions	Five-point Likert scores^a^			
	Expert 1	Expert 2	Expert 3	Median
1. Which patients need to consider combined thoracolumbar spinal cord injury?	5	5	4	5
2. How to immobilize, transport, and transfer patients with suspected thoracolumbar spinal cord injury?	5	5	5	5
3. How to assess the degree of neurological injury in patients with acute thoracolumbar spinal cord injury?	5	5	4	5
4. How to perform radiological assessment for patients with suspected acute thoracolumbar spinal cord injury?	4	4	4	4
5. How to assess the morphology of injury in patients with acute thoracolumbar spinal cord injury?	4	4	4	4
6. Is the use of high-dose corticosteroids and gangliosides recommended for the treatment of spinal cord injury?	5	5	5	5
7. What are the indications for the treatment selection in patients with acute thoracolumbar spinal cord injury?	5	5	5	5
8. What are the recommended conservative treatment methods for thoracolumbar spinal cord injury?	5	5	5	5
9. What is the timing for surgery in patients with acute thoracolumbar spinal cord injury?	4	5	4	4
10. How to choose the surgical approach for patients with acute thoracolumbar spinal cord injury?	5	5	4	5
11. When is laminectomy necessary for patients with acute thoracolumbar spinal cord injury?	5	5	5	5
12. How to select the fixation segment for posterior surgery in patients with acute thoracolumbar spinal cord injury?	5	5	5	5
13. Is it necessary to fix the injured vertebrae posteriorly in patients with acute thoracolumbar spinal cord injury?	4	5	4	4
14. What are the indications for percutaneous internal fixation in patients with acute thoracolumbar spinal cord injury?	5	5	5	5
15. Is it necessary to perform bone graft fusion for patients with acute thoracolumbar spinal cord injury who undergo surgery?	5	4	5	5
16. Is simple pedicle-based bone grafting effective in patients with acute thoracolumbar spinal cord injury?	5	5	5	5
17. How to manage the urinary system in patients with acute thoracolumbar spinal cord injury?	5	5	5	5
18. How to prevent and treat deep vein thrombosis in patients with acute thoracolumbar spinal cord injury?	5	5	5	5
19. Is it necessary to prevent and treat pressure sores in patients with acute thoracolumbar spinal cord injury?	5	5	5	5
20. How to manage neurogenic bowel in patients with acute thoracolumbar spinal cord injury?	5	5	5	5
21. Does early surgical intervention improve outcomes for patients with thoracic and lumbar fractures?	4	5	4	4
22. Does the choice of surgical approach (anterior, posterior, or combined anterior-posterior) improve clinical outcomes in patients with thoracic and lumbar fractures?	2	2	2	2
23. Are there radiographic findings in patients with traumatic thoracolumbar fractures that can predict the need for surgical intervention?	5	5	5	5
24. Are there radiographic findings in patients with traumatic thoracolumbar fractures that can assist in predicting clinical outcomes?	3	4	3	3
25. Does routine screening for DVT prevent PE (or VTE-associated morbidity and mortality) in patients with thoracic and lumbar fractures?	5	5	5	5
26. For patients with thoracic and lumbar fractures, is one regimen of VTE prophylaxis superior to others with respect to prevention of PE (or VTE-associated morbidity and mortality)?	5	5	5	5
27. Is there a specific treatment regimen for documented VTE that provides fewer complications than other treatments in patients with thoracic and lumbar fractures?	4	4	4	4
28. Does the administration of a specific pharmacologic agent (e.g., methylprednisolone) improve clinical outcomes in patients with thoracic and lumbar fractures and spinal cord injury?	5	5	5	5
29. Does the surgical treatment of burst fractures of the thoracic and lumbar spine improve clinical outcomes compared to nonoperative treatment?	4	5	5	5
30. Does the surgical treatment of nonburst fractures of the thoracic and lumbar spine improve clinical outcomes compared to nonoperative treatment?	4	5	4	4
31. Does the addition of arthrodesis to instrumented fixation improve outcomes in patients with thoracic and lumbar burst fractures?	5	5	5	5
32. How does the use of minimally invasive techniques (including percutaneous instrumentation) affect outcomes in patients undergoing surgery for thoracic and lumbar fractures compared to conventional open techniques?	5	5	5	5
33. Does the use of external bracing improve outcomes in the nonoperative treatment of neurologically intact patients with thoracic and lumbar burst fractures?	5	5	5	5
34. Which neurological assessment tools have demonstrated internal reliability and validity in the management of patients with thoracic and lumbar fractures (i.e., do these instruments provide consistent information between different care providers)?	4	5	4	4
35. Are there any clinical findings (e.g., presenting neurological grade/function) in patients with thoracic and lumbar fractures that can assist in predicting clinical outcomes?	5	5	5	5
36. Does the active maintenance of arterial blood pressure after injury affect clinical outcomes in patients with thoracic and lumbar fractures?	5	5	5	5
37. Are there classification systems for fractures of the thoracolumbar spine that have been shown to be internally valid and reliable (i.e., do these instruments provide consistent information between different care providers)?	5	4	5	5
38. In treating patients with thoracolumbar fractures, does employing a formally tested classification system for treatment decision-making affect clinical outcomes?	5	5	5	5

DVT, deep vein thrombosis; PE, pulmonary embolism.

^a^ Five-point Likert score system: 1 means completely incorrect; 2 means more incorrect than correct; 3 means equally incorrect and correct; 4 means more correct than incorrect; 5 means completely correct.

## Discussion

When asked, “Does the choice of surgical approach (anterior, posterior, or combined anterior-posterior) improve clinical outcomes in patients with thoracic and lumbar fractures?”, ChatGPT4o provided an affirmative answer along with detailed explanations. However, according to CNS guidelines, for patients with burst fractures of the thoracolumbar spine, surgeons may use an anterior, posterior, or combined approach, as the choice of approach does not significantly affect clinical or neurological outcomes, a Grade B recommendation. Although ChatGPT4o provided a detailed explanation of the indications for each approach, the experts noted that while the response was generally accurate, the final conclusion was not entirely consistent with guideline recommendations. Furthermore, while ChatGPT4o appears capable of conducting targeted searches on open websites, its “independent reasoning” abilities require further refinement.

In summary, ChatGPT4o demonstrates promising performance in diagnosing and treating thoracolumbar trauma. Its ability to search open websites and provide detailed responses could be a useful reference for clinical practitioners. However, ChatGPT4o does not consistently provide fully accurate answers, particularly with “yes or no” questions. Its dependence on specific sources for data retrieval may introduce biases that limit its broader application in the field of spine surgery. ChatGPT requires substantial medical data for further training to enhance model performance. Moreover, given the specific ethical considerations in medicine, ChatGPT4o's use in clinical settings must ensure patient safety, data privacy, ethical standards, and adherence to relevant “AI regulations”. Although ChatGPT4o's responses may improve clinical efficiency, it should only serve as a clinical assistant, with spine surgeons validating the accuracy of its information.

This study has several methodological limitations: firstly, the lack of comparative analyses with established AI systems (e.g., Google Med-PaLM, IBM Watson) or traditional decision-support tools hinders definitive performance benchmarking; secondly, simulated testing environments may overestimate system efficacy, as diagnostic performance degradation in real-world clinical settings requires urgent empirical validation; finally, the rapid evolution of AI technology necessitates dynamically updated training databases and ethical evaluation frameworks. To address these gaps, subsequent research will incorporate the Partial Credit Model (PCM) and Item Response Theory (IRT) through latent trait modeling, systematically quantifying AI response difficulty levels, refining multidimensional scoring criteria, and strengthening clinical applicability assessments to establish a psychometrically-based evaluation framework. This methodological advancement will enhance the granular understanding of AI's role in complex medical decision-making (e.g., surgical approach selection, prognostic stratification). Future research priorities include: (1) comparative effectiveness studies across AI systems, (2) real-world clinical validation of performance, and (3) development of specialty-specific human-AI collaboration guidelines to systematically improve the clinical utility of intelligent assistive tools in spinal surgery.
