# Supplementary material for: ChatGPT4o's theranostic performance in the management of thoracolumbar spine fractures
Source: Front Surg. 2025 Feb 25;12:1524396. doi: 10.3389/fsurg.2025.1524396 (PMC11893555; doi:10.3389/fsurg.2025.1524396)
Supplement: Supplementary file 1 [file Datasheet1.pdf]

<table>
<tr><td>Questions</td><td>Answers</td></tr>
<tr><td>1. Which patients need to consider combined thoracolumbar spinal cord injury?</td><td>Combined thoracolumbar spinal cord injury should be considered in patients with:
1. Multilevel Vertebral Fractures: When fractures extend across the thoracic and lumbar vertebrae, especially involving T10-L2, as this area marks the thoracolumbar junction where mobility and weight-bearing shift between the thoracic and lumbar spine.
2. Neurological Deficits: Patients presenting with significant neurological impairments, such as motor weakness, sensory loss, or bowel/bladder dysfunction, may need evaluation for combined spinal cord injuries, as damage can span both thoracic and lumbar segments.
3. High-Impact Trauma: Those who have sustained severe trauma (e.g., high-speed vehicle accidents, falls from significant heights) are at higher risk for combined injuries, given the force required to damage multiple vertebral levels.
4. Concomitant Spinal Instability: Patients with both ligamentous injury and bony disruption in the thoracolumbar region might need surgical consideration to stabilize the combined injury and prevent worsening of spinal cord damage.
5. Underlying Spinal Conditions: Those with conditions like osteoporosis, spinal tumors, or deformities (e.g., scoliosis, kyphosis) that weaken the structural integrity of the spine may need more extensive evaluations, as they are predisposed to sustaining multiple injuries in a single traumatic event.
Evaluating these patients requires a detailed imaging study, such as an MRI or CT scan, to assess the full extent of injury, stability, and any neural compromise in both thoracic and lumbar regions.</td></tr>
<tr><td>2. How to immobilize, transport, and transfer patients with suspected thoracolumbar spinal cord injury?</td><td>For patients with a suspected thoracolumbar spinal cord injury, proper immobilization, transport, and transfer are crucial to prevent further spinal cord damage. Here are the recommended steps:
1. Immobilization
• Use of Cervical Collar: Although thoracolumbar injuries are below the cervical spine, a cervical collar may be applied initially to prevent additional movement in cases where the full extent of spinal injury is uncertain.
• Spinal Immobilization: Position the patient on a rigid backboard or a vacuum mattress to limit movement of the spine. Ensure the patient’s body is kept in a neutral, aligned position, especially at the thoracolumbar junction.
• Straps or Padding: Secure the patient with straps across the chest, hips, and legs to minimize motion during transport.</td></tr>
</table>

	Padding may be used to fill gaps and prevent excessive movement.<br>**2. Transport**<br>• **Log Roll Technique**: Use the **log roll maneuver** if necessary to position the patient on the backboard. This requires a coordinated effort from at least three rescuers (one to support the head and neck, two to roll the body in unison) to ensure the spine remains aligned.<br>• **Head and Torso Stability**: Maintain support at the head, shoulders, and pelvis while moving the patient to prevent any twisting or bending at the thoracolumbar junction.<br>• **Ambulance Transport**: Transport the patient using an ambulance with an **adequately secured backboard** to prevent shifting. During transit, monitor for any changes in neurological status and continue to support the spine.<br>**3. Transfer to Hospital Bed**<br>• **Slide Transfer Method**: At the hospital, transfer the patient from the backboard to the hospital bed using a **sliding board or sheet** transfer. This method requires multiple team members to lift and slide the patient in a controlled, synchronized manner.<br>• **Avoid Lifting Directly**: Avoid lifting the patient directly, as this can cause unintentional flexion or rotation of the spine. A **transfer board** reduces the risk of spinal movement during the transfer.<br>• **Continued Immobilization**: Once transferred to the hospital bed, ensure the patient remains immobilized until further imaging confirms the injury level and treatment plan.<br>Proper immobilization, careful handling, and coordinated teamwork are essential throughout these steps to prevent any exacerbation of the spinal cord injury.
3. How to assess the degree of neurological injury in patients with acute thoracolumbar spinal cord injury?	Assessing the degree of neurological injury in patients with an acute thoracolumbar spinal cord injury requires a systematic approach to evaluate sensory, motor, and functional impairments. The **American Spinal Injury Association (ASIA) Impairment Scale (AIS)** is a widely used tool for this purpose, as it provides a structured assessment and classification of the injury. Here's how to perform the assessment:<br>**1. Initial Assessment: ASIA Impairment Scale (AIS)**<br>The ASIA Impairment Scale assesses sensory and motor function in key areas:<br>• **Sensory Exam**:

	○ Evaluate **light touch** and **pinprick sensation** at each dermatome (sensory segment) from T1 to S5.<br>○ Record responses as normal, impaired, or absent at each level.<br>○ Check for **sacral sensation** (at S4-S5), including perianal sensation and voluntary anal contraction, as sacral sparing can indicate an incomplete injury.<br>• **Motor Exam**:<br>○ Assess motor strength in key muscle groups corresponding to specific thoracic and lumbar levels.<br>○ Grade muscle strength on a scale from 0 (no movement) to 5 (normal strength) using standard manual muscle testing.<br>○ Key muscle groups for thoracolumbar evaluation include hip flexors (L2), knee extensors (L3), ankle dorsiflexors (L4), and big toe extensors (L5).<br>**2. ASIA Impairment Scale Classification**<br>After completing the sensory and motor exams, classify the injury level according to the ASIA Impairment Scale:<br>• **AIS A**: Complete injury with no motor or sensory function preserved below the level of injury, including sacral segments.<br>• **AIS B**: Incomplete injury with sensory but not motor function preserved below the level of injury, including sacral segments.<br>• **AIS C**: Incomplete injury with motor function preserved below the level of injury, and more than half of the key muscles below the injury level have a muscle grade of less than 3.<br>• **AIS D**: Incomplete injury with motor function preserved below the level of injury, and at least half of the key muscles below the injury level have a muscle grade of 3 or higher.<br>• **AIS E**: Normal sensory and motor function.<br>**3. Additional Assessments**<br>• **Rectal Tone and Voluntary Anal Contraction**: Check rectal tone and assess for voluntary anal contraction, as sacral sparing indicates an incomplete injury.<br>• **Deep Tendon Reflexes**: Evaluate reflexes in the lower extremities, as absent or abnormal reflexes may correlate with specific spinal cord levels.<br>• **Spinal Shock**: Determine if the patient is experiencing spinal shock, characterized by flaccid paralysis and absence of reflexes, which may temporarily obscure the level and completeness of injury. This typically resolves over days to weeks.

	**4. Imaging and Advanced Diagnostics**<br>• **MRI**: Conduct MRI to visualize the spinal cord, identify compression, hemorrhage, edema, or other structural changes that may indicate the severity and extent of injury.<br>• **CT and X-ray**: For detailed bony assessment and alignment, CT or X-ray imaging is often performed to determine the degree of vertebral injury and stability.<br>**5. Functional Assessment**<br>• **Functional Independence Measure (FIM)**: Assess the patient's ability to perform daily activities, which helps gauge functional impairment and aids in rehabilitation planning.<br>• **Bladder and Bowel Function**: Evaluate bladder and bowel function as thoracolumbar injuries frequently affect autonomic control, impacting sphincter control and continence.<br>This structured approach to neurological assessment using the ASIA Scale, combined with imaging and functional evaluations, allows for an accurate diagnosis and classification of the injury, guiding management and rehabilitation.
4. How to perform radiological assessment for patients with suspected acute thoracolumbar spinal cord injury?	A thorough radiological assessment is essential for diagnosing and determining the severity of acute thoracolumbar spinal cord injury. This assessment typically involves a combination of X-ray, CT, and MRI imaging to evaluate both bony and soft tissue structures. Here's how to perform a radiological assessment:<br>**1. Initial X-ray Imaging**<br>• **Purpose**: X-rays are often the first-line imaging modality to quickly assess spinal alignment, fractures, and dislocations.<br>• **Views**:<br>○ Obtain **anteroposterior (AP)** and **lateral** views of the thoracolumbar spine.<br>○ **Oblique views** may help identify facet joint injuries.<br>• **Focus Areas**:<br>○ Check for **vertebral body height loss**, **fracture lines**, **malalignment**, or **widening of interspinous spaces**.<br>○ Look for signs of **spinous process fractures** or **pedicle fractures**, which may indicate instability.<br>**2. CT Scan (Computed Tomography)**<br>• **Purpose**: CT provides high-resolution images of bony structures and is highly effective for detecting fractures, fragment displacements, and complex bony injuries.

	• **Protocol**:<br>○ Perform a **CT scan of the entire thoracolumbar region** to capture any injuries that may have been missed on X-ray.<br>○ **Multiplanar reconstructions** (sagittal and coronal) help in evaluating fractures in multiple planes.<br>• **Focus Areas**:<br>○ Evaluate for **compression fractures, burst fractures, vertebral body fractures**, and **retropulsed fragments** that may impinge on the spinal canal.<br>○ Assess for **intervertebral disc involvement**, which may indicate the need for surgical intervention if the disc has herniated.<br>**3. MRI (Magnetic Resonance Imaging)**<br>• **Purpose**: MRI is the gold standard for assessing soft tissue, including the spinal cord, ligaments, intervertebral discs, and paraspinal structures. It is essential for determining the extent of spinal cord damage, edema, or hemorrhage.<br>• **Protocol**:<br>○ Obtain **T1-weighted**, **T2-weighted**, and **STIR (Short Tau Inversion Recovery)** sequences to identify soft tissue injuries.<br>○ Focus on **sagittal** and **axial** views to evaluate cord integrity and soft tissue involvement.<br>• **Focus Areas**:<br>○ Look for **spinal cord compression, edema, hemorrhage, ligamentous injury**, and **disc herniation**.<br>○ Evaluate the extent of **spinal cord injury** (e.g., edema or myelomalacia) and check for signs of **cord transection** if suspected.<br>○ Identify any **posterior ligament complex (PLC)** injuries, as these are critical for stability and often indicate the need for surgical intervention.<br>**4. Additional Imaging Considerations**<br>• **Flexion-Extension X-rays**: In cases where spinal instability is suspected but not clear on static images, dynamic flexion-extension X-rays can help. However, these are typically performed only once the patient is stabilized and under the guidance of a specialist, as they carry a risk of further injury.

	• **Whole-Body CT**: For patients with polytrauma or high-impact injuries, a whole-body CT scan may be appropriate to assess for concurrent injuries in other areas, as thoracolumbar injuries are commonly associated with trauma to other body parts.<br>**5. Grading and Classification**<br>• Use a spinal injury classification system, such as the **Thoracolumbar Injury Classification and Severity Score (TLICS)**, which incorporates radiological findings to guide treatment:<br>○ **Injury morphology**: compression, burst, translational/rotational, or distraction injuries.<br>○ **PLC integrity**: intact, indeterminate, or disrupted.<br>○ **Neurological status**: intact, nerve root injury, complete or incomplete spinal cord injury.<br>• The TLICS score can aid in determining if a conservative or surgical approach is necessary.<br>**6. Imaging Follow-Up**<br>• **Repeat Imaging**: In some cases, repeat MRI or CT imaging may be necessary to monitor the progression of edema, hemorrhage, or other changes, especially if there is a neurological deterioration.<br>• **Preoperative Imaging**: For patients requiring surgery, preoperative imaging with CT and MRI helps in surgical planning, ensuring precise knowledge of fracture anatomy and soft tissue status.<br>This comprehensive radiological assessment helps in identifying the type and extent of thoracolumbar injury, guiding treatment decisions, and determining the prognosis for recovery.
5. How to assess the morphology of injury in patients with acute thoracolumbar spinal cord injury?	Assessing the morphology of injury in patients with acute thoracolumbar spinal cord injury is essential to understanding the type, extent, and severity of the injury, which informs the treatment approach and prognosis. Key aspects of injury morphology include fracture pattern, spinal alignment, and soft tissue integrity. Here's how to systematically assess injury morphology:<br>**1. Identify Fracture Patterns**<br>• **Compression Fractures**:<br>○ These involve the collapse of the anterior part of the vertebral body.<br>○ Often stable, but if the compression is severe, it can indicate instability.<br>○ Seen commonly in flexion injuries, they present as a wedge-shaped vertebra on lateral imaging.<br>• **Burst Fractures**:<br>○ Result from high-energy axial loading, causing the vertebral body to shatter.

- Fragments may retropulse into the spinal canal, causing spinal cord compression.
- Assess for canal compromise and neurological deficits due to potential instability.

- **Chance Fractures (Flexion-Distraction Injuries)**:
  - Often associated with seatbelt injuries, they result from flexion-distraction forces.
  - The injury usually involves the vertebral body, pedicles, and posterior elements.
  - Typically unstable due to disruption of the posterior ligamentous complex.
- **Fracture-Dislocation Injuries**:
  - These occur from translational or rotational forces, causing displacement of one vertebra over another.
  - High risk of spinal cord injury and usually involves extensive ligamentous disruption.
  - These are generally unstable injuries requiring surgical stabilization.

2. Assess Vertebral Alignment and Stability

- **Spinal Alignment**:
  - Evaluate alignment in the **sagittal**, **coronal**, and **axial planes** on imaging (X-ray, CT, or MRI).
  - Look for step-offs, translation, or angulation at the injury site, which may indicate instability.
- **Posterior Ligamentous Complex (PLC) Integrity**:
  - Assess the posterior ligamentous complex, which includes the supraspinous ligament, interspinous ligament, ligamentum flavum, and facet joint capsules.
  - MRI is ideal for evaluating PLC integrity, as injuries here often indicate instability.
  - Disruption of the PLC usually necessitates surgical intervention, as it's crucial for spine stability.

3. Evaluate the Degree of Spinal Canal Compromise

- **Canal Compression**:
  - In burst fractures and fractures with retropulsed fragments, assess the degree of spinal canal narrowing.
  - A **CT scan** with axial cuts provides detailed visualization of bony fragments encroaching on the canal.
  - Quantify the percentage of canal compromise to help gauge the risk of neurological injury.
- **Spinal Cord and Nerve Root Compression**:
  - Use **MRI** to evaluate direct spinal cord or nerve root compression due to retropulsed fragments, hematoma, or

	disc herniation.<br>◦ Identify any areas of cord edema, hemorrhage, or myelomalacia, as these indicate the extent of cord damage.<br>**4. Assess for Discs and Facet Joint Involvement**<br>• **Disc Integrity**:<br>◦ Assess intervertebral discs for herniation, which can accompany burst and flexion injuries.<br>◦ Look for **disc herniation** that may impinge on the spinal cord or nerve roots, visible on MRI.<br>• **Facet Joint Integrity**:<br>◦ Evaluate facet joints on CT and MRI for dislocation or fracture.<br>◦ Facet dislocation indicates instability and often requires surgical stabilization.<br>**5. Classification Using TLICS**<br>The **Thoracolumbar Injury Classification and Severity Score (TLICS)** is a tool that can be used to categorize the morphology of thoracolumbar injuries. It takes into account:<br>• **Injury Morphology** (e.g., compression, burst, translational/rotational injuries).<br>• **Posterior Ligamentous Complex Integrity** (intact, indeterminate, disrupted).<br>• **Neurological Status** (intact, nerve root injury, complete or incomplete cord injury).<br>Each category is assigned a score, with a total score guiding the decision between conservative and surgical treatment.<br>**6. Follow-Up Imaging for Morphology Assessment**<br>• In some cases, repeat imaging (usually MRI) may be necessary to monitor for changes in spinal alignment, disc position, or the progression of cord injury.<br>• **Dynamic Imaging**: Flexion-extension X-rays are used only in cases where instability is suspected after initial imaging and are done under careful medical supervision.<br>This comprehensive assessment of injury morphology provides critical insights into the type and severity of thoracolumbar injury and helps guide treatment decisions for optimal outcomes.
6. Is the use of high-dose corticosteroids and gangliosides recommended for the treatment of spinal cord injury?	The use of high-dose corticosteroids and gangliosides in the treatment of spinal cord injury (SCI) has been extensively studied, but their efficacy and safety remain subjects of debate.<br>**High-Dose Corticosteroids:**

	Historically, high-dose methylprednisolone was administered to acute SCI patients based on findings from the National Acute Spinal Cord Injury Study (NASCIS) trials, which suggested potential neurological benefits when administered within eight hours of injury.<br>However, subsequent analyses have raised concerns about the associated risks, including increased susceptibility to infections and other complications.<br>As a result, the routine use of high-dose corticosteroids in acute SCI is now controversial, with many guidelines advising against their standard application due to the uncertain balance between potential benefits and harms.<br>**Gangliosides:**<br>Gangliosides, particularly monosialo-tetrahexosyl-ganglioside (GM1), have been investigated for their neuroprotective properties in SCI. Some studies have reported faster recovery rates in patients treated with GM1 compared to placebo.<br>Despite these findings, the clinical use of gangliosides has been limited due to concerns about safety, inconsistent results across studies, and the lack of robust, large-scale clinical trials confirming their efficacy.<br>**Current Recommendations:**<br>Given the mixed evidence and potential risks, neither high-dose corticosteroids nor gangliosides are universally recommended for the treatment of SCI. Clinical decisions should be individualized, considering the specific circumstances of each patient and the most current clinical guidelines. Ongoing research continues to explore alternative therapies and interventions aimed at improving outcomes for individuals with SCI.
7. What are the indications for the treatment selection in patients with acute thoracolumbar spinal cord injury?	Treatment selection for patients with acute thoracolumbar spinal cord injury (SCI) depends on several key indications that help guide whether conservative or surgical management is more appropriate. Here are the main factors to consider:<br>**1. Neurological Status**<br>• **Complete vs. Incomplete Injury**: Patients with complete SCI (no sensory or motor function below the level of injury) may have less to gain from surgical decompression compared to those with incomplete injuries, where some preservation of function exists.<br>• **Spinal Cord Compression**: Patients with neurological deficits due to spinal cord or nerve root compression often benefit from surgical decompression.<br>**2. Stability of the Injury**<br>• **Unstable Fractures**: Fractures involving disruption of the posterior ligamentous complex, burst fractures with retropulsed

	fragments, or fractures with significant displacement are generally unstable and often require surgical intervention to restore stability.<br>• **Stable Fractures**: Stable fractures, such as minor compression fractures without posterior ligamentous disruption, can typically be managed conservatively with bracing and monitoring.<br>**3. Type of Fracture and Morphology**<br>• **Compression Fractures**: Usually considered stable and may not require surgery unless there is a high degree of compression or loss of vertebral body height.<br>• **Burst Fractures**: Depending on canal compromise and neurological symptoms, burst fractures may require surgical intervention, especially if there are retropulsed fragments.<br>• **Chance Fractures and Fracture-Dislocations**: Flexion-distraction and translational/rotational injuries are often unstable and usually require surgical stabilization.<br>**4. Patient's General Health and Comorbidities**<br>• **Age and Comorbidities**: Older patients or those with significant comorbidities may have higher surgical risks, leading to consideration of non-surgical options if the injury is stable.<br>• **Preexisting Conditions**: Conditions such as osteoporosis or previous spinal surgery may influence the choice of treatment, as these patients may require more stabilization.<br>**5. Pain and Functional Considerations**<br>• **Severe Pain**: Persistent or severe pain, particularly if refractory to conservative management, may indicate the need for surgical intervention.<br>• **Functional Goals**: The potential for functional recovery, especially in cases of incomplete injury, may favor surgical decompression and stabilization to maximize the patient's rehabilitation potential.<br>**6. Patient Preferences and Rehabilitation Potential**<br>• **Patient's Functional Goals and Lifestyle**: For patients with high functional demands or a desire for early mobilization, surgical intervention may be preferred to facilitate more rapid rehabilitation.<br>• **Rehabilitation Requirements**: Patients with incomplete injuries or those undergoing rehabilitation may benefit from surgical stabilization to allow safer mobilization and physical therapy.

	**7. Scoring Systems and Classification**<br>• **Thoracolumbar Injury Classification and Severity Score (TLICS)**: This scoring system evaluates injury morphology, posterior ligamentous complex integrity, and neurological status to guide treatment. Higher scores indicate the need for surgery, while lower scores suggest conservative management may be appropriate.<br>**Summary of Treatment Selection**<br>• **Surgical Management**: Generally indicated for patients with unstable fractures, neurological deficits due to compression, incomplete injuries with potential for recovery, or significant canal compromise.<br>• **Conservative Management**: Indicated for patients with stable fractures, minimal or no neurological deficits, and high surgical risk due to comorbidities, provided pain and function can be managed without surgical intervention.<br>Treatment decisions should be individualized based on these indications, with multidisciplinary input and consideration of patient-specific factors to optimize outcomes.
8. What are the recommended conservative treatment methods for thoracolumbar spinal cord injury?	Conservative treatment is an option for stable thoracolumbar spinal cord injuries (SCI) or for patients who are at high surgical risk. Here are the main conservative treatment methods recommended for thoracolumbar SCI:<br>**1. Spinal Immobilization and Bracing**<br>• **Braces**: The use of orthotic braces, such as a **thoracolumbar-sacral orthosis (TLSO)** or a **Jewett brace**, helps maintain spinal alignment and stability.<br>○ **TLSO** provides circumferential support from the thoracic spine to the sacrum, limiting motion in all planes.<br>○ **Jewett brace** primarily restricts flexion, which is useful for certain types of compression fractures.<br>• **Duration of Bracing**: Bracing is typically recommended for **8–12 weeks**, depending on the severity and type of fracture, and is gradually reduced as the spine heals.<br>• **Patient Education**: Teach patients proper use and maintenance of the brace, including wearing time and adjustment, to ensure effective stabilization.<br>**2. Bed Rest and Gradual Mobilization**<br>• **Initial Bed Rest**: Short-term bed rest (a few days) may be recommended initially to allow swelling and inflammation to subside, especially if there is acute pain or neurological irritation.<br>• **Gradual Mobilization**: Early mobilization, with or without a brace, is encouraged as pain improves to avoid complications

	from prolonged bed rest, such as muscle atrophy, deep vein thrombosis, and pressure ulcers.<br>• **Weight-Bearing Restrictions**: Patients should avoid heavy lifting or bending motions that could put stress on the injured area. Movement is reintroduced based on the stability of the injury and pain levels.<br>**3. Pain Management**<br>• **Medications**:<br>○ **Nonsteroidal Anti-Inflammatory Drugs (NSAIDs)**: Used to manage pain and inflammation.<br>○ **Acetaminophen**: A milder option for pain control, often used in combination with other medications.<br>○ **Muscle Relaxants**: Prescribed for muscle spasms, which are common after SCI.<br>○ **Opioids**: Reserved for severe pain and generally used for short-term relief.<br>• **Neuropathic Pain Medications**: For cases with nerve pain, gabapentin or pregabalin may be recommended.<br>**4. Physical Therapy and Rehabilitation**<br>• **Strengthening Exercises**: Focus on strengthening the core and back muscles to improve stability and support around the injury site.<br>• **Flexibility Exercises**: Gentle stretching can improve mobility without placing stress on the spine.<br>• **Functional Training**: Training is often customized to help patients perform daily activities safely and independently.<br>• **Gradual Increase in Activity**: Under the supervision of a physical therapist, patients gradually increase their activity level as pain subsides and stability improves.<br>**5. Occupational Therapy**<br>• **Adaptive Techniques**: Occupational therapists work with patients to adapt daily activities, minimizing strain on the spine.<br>• **Education on Body Mechanics**: Patients learn safe movement techniques, including proper posture, lifting, and bending, to protect the spine during recovery.<br>**6. Close Monitoring and Follow-Up**<br>• **Regular Imaging**: Follow-up X-rays, CT scans, or MRIs may be scheduled to monitor fracture healing and ensure there is no worsening of alignment or stability.<br>• **Neurological Monitoring**: Regular assessments of neurological function help track recovery or detect any changes.<br>• **Adjusting Treatment as Needed**: Based on follow-up evaluations, bracing may be adjusted or discontinued, and activity

	restrictions modified as healing progresses.<br>**7. Lifestyle Modifications**<br>• **Avoiding High-Risk Activities**: Patients are advised to avoid activities that pose a high risk of re-injury, such as heavy lifting, contact sports, or high-impact exercises.<br>• **Bone Health**: For patients with osteoporosis or other bone health issues, treatments may include calcium and vitamin D supplements, along with weight-bearing exercises, to strengthen bones.<br>Conservative treatment requires adherence to prescribed activity restrictions, physical therapy, and regular monitoring to ensure healing and prevent complications. A collaborative approach involving the physician, physical therapist, occupational therapist, and patient is essential to achieve optimal recovery and maintain spinal stability.
9. What is the timing for surgery in patients with acute thoracolumbar spinal cord injury?	The timing of surgery in patients with acute thoracolumbar spinal cord injury (SCI) depends on several factors, including the severity of the injury, neurological status, and presence of spinal instability or compression. Here is an overview of considerations for surgical timing:<br>**1. Immediate Surgery (Within 24 Hours)**<br>• **Indications**: Immediate surgery is generally indicated for patients with:<br>○ Progressive neurological deficits<br>○ Significant spinal cord or nerve root compression<br>○ Unstable fractures with potential for worsening alignment<br>○ Fracture-dislocation injuries with vertebral displacement<br>• **Rationale**: Early decompression, within the first 24 hours, may improve neurological outcomes by relieving spinal cord compression and stabilizing the spine, allowing for safer mobilization and rehabilitation.<br>**2. Early Surgery (Within 72 Hours)**<br>• **Indications**: For patients with incomplete SCI or stable neurological status but with evidence of cord compression or instability, surgery within 24–72 hours is recommended.<br>• **Rationale**: Early surgery is associated with lower rates of complications, shorter hospital stays, and potentially better functional outcomes, especially in patients with incomplete injuries. Studies suggest that early decompression may reduce inflammation and secondary injury to the spinal cord.

	**3. Delayed Surgery (After 72 Hours)**<br>• **Indications**: Delayed surgery may be considered in:<br>○ Patients with stable fractures or injuries not causing neurological compromise<br>○ Patients with medical conditions that preclude immediate surgery (e.g., hemodynamic instability, coagulopathies)<br>○ Cases where conservative management has failed, or neurological deterioration occurs<br>• **Rationale**: Delayed surgery may be necessary to allow medical stabilization or to manage other injuries before addressing the spinal injury. It is generally less effective for recovery of neurological function but can be essential for stabilizing the spine and managing pain or deformities in the longer term.<br>**4. Factors Influencing Surgical Timing**<br>• **Neurological Status**: Patients with incomplete injuries tend to benefit more from early decompression. For complete injuries, early surgery may still be beneficial for stabilization but may not affect neurological recovery.<br>• **Type of Fracture**: Highly unstable fractures, such as burst fractures with retropulsed fragments or fracture-dislocations, require urgent intervention to prevent further neurological injury.<br>• **Overall Health and Stability**: For patients with polytrauma or other life-threatening injuries, prioritizing stabilization of other critical systems may delay spinal surgery.<br>**5. Benefits of Early Surgery**<br>• Improved neurological outcomes, particularly in incomplete injuries<br>• Reduced complications, such as respiratory infections and pressure sores, associated with prolonged immobility<br>• Shorter hospital stays and faster initiation of rehabilitation<br>**Summary**<br>Early surgery (within 24–72 hours) is generally recommended for patients with acute thoracolumbar SCI, especially those with incomplete injuries and spinal cord compression, as it can lead to better neurological and functional outcomes. However, the exact timing should be individualized based on the patient's medical condition, injury stability, and neurological presentation.
10. How to choose the surgical approach for patients with acute thoracolumbar spinal cord injury?	Choosing the appropriate surgical approach for patients with acute thoracolumbar spinal cord injury (SCI) is a critical decision that depends on the type and location of the injury, stability, presence of neural compression, and individual patient factors. Here are key considerations for selecting the optimal surgical approach:

	**1. Posterior Approach**<br>• **Indications**:<br>○ **Posterior ligamentous complex (PLC) injury**: When the posterior stabilizing ligaments or facet joints are disrupted.<br>○ **Spinal instability**: To provide stabilization through instrumentation and fusion (screws and rods).<br>○ **Fracture-dislocation injuries**: Especially useful when there is significant displacement or rotational deformity.<br>○ **Multilevel Stabilization**: The posterior approach allows access to multiple levels, making it effective for long-segment stabilization.<br>• **Benefits**:<br>○ Provides excellent stabilization with screws and rods, helping to prevent further spinal deformity.<br>○ Allows indirect decompression of the spinal canal by reducing fracture fragments.<br>○ Often preferred due to the simpler surgical setup and lower risk of vascular injury compared to anterior approaches.<br>• **Limitations**:<br>○ Less effective for direct decompression of retropulsed fragments pressing on the spinal cord.<br>○ May require extensive muscle dissection, which can lead to increased postoperative pain and longer recovery times.<br>**2. Anterior Approach**<br>• **Indications**:<br>○ **Severe burst fractures** with retropulsed bony fragments compressing the spinal cord or cauda equina.<br>○ **Direct decompression** needs: For injuries where anterior structures (e.g., vertebral body or disc) are the main source of neural compression.<br>○ **Single-level injury**: The anterior approach is often preferred for isolated injuries that need decompression and stabilization at one or two levels.<br>• **Benefits**:<br>○ Allows direct access to the vertebral body and disc, facilitating removal of retropulsed fragments and effective

	decompression of the spinal cord.<br>◦ Can achieve better vertebral body reconstruction and height restoration with the use of cages or grafts.<br>◦ Generally results in less postoperative pain and faster recovery compared to posterior approaches due to reduced muscle disruption.<br>• **Limitations**:<br>◦ More technically challenging and carries risks of injury to major vascular structures (e.g., aorta, vena cava) and visceral organs.<br>◦ Limited access to posterior structures, making it less suitable for cases requiring extensive stabilization across multiple levels.<br>**3. Combined Anterior and Posterior Approach**<br>• **Indications**:<br>◦ **Complex injuries**: For severe burst fractures or fracture-dislocations requiring both extensive decompression and stabilization.<br>◦ **Instability with neural compression**: In cases where both anterior decompression and posterior stabilization are essential.<br>◦ **Failed single approach**: When one approach alone is inadequate to achieve stability and decompression.<br>• **Benefits**:<br>◦ Provides the most comprehensive access to both anterior and posterior elements, allowing direct decompression of the spinal canal and robust stabilization.<br>◦ Can improve alignment and restore spinal stability in highly unstable or complex fractures.<br>• **Limitations**:<br>◦ Longer surgery time, greater blood loss, and increased risk of postoperative complications.<br>◦ Often requires staged surgeries or a longer recovery period due to the extensive nature of the approach.<br>**4. Minimally Invasive Surgery (MIS)**<br>• **Indications**:<br>◦ **Stable or less severe fractures**: Minimally invasive options are suitable for patients who need limited stabilization

or decompression.

  - **Medical comorbidities**: When traditional open surgery carries high risks, MIS can be a less invasive alternative.
  - **Burst fractures without significant retropulsion**: If fragments are stable and do not press on the spinal cord, percutaneous fixation may suffice.
- **Benefits**:
  - Reduced blood loss, muscle damage, and postoperative pain.
  - Shorter hospital stays and faster recovery.
- **Limitations**:
  - Limited ability to achieve extensive decompression, which may not be suitable for cases with severe spinal canal compromise.
  - Technically demanding and requires specialized equipment and expertise.

5. Factors to Consider When Choosing the Surgical Approach

- **Type of Fracture and Stability**: Burst fractures with retropulsed fragments often require anterior decompression, while fractures with posterior instability may need a posterior or combined approach.
- **Neurological Status**: Severe compression with neurological deficits often requires a more direct decompressive approach, such as anterior or combined methods.
- **Surgeon's Expertise and Facility Resources**: Some approaches require specific expertise and may only be feasible in centers with the appropriate equipment and experience.

Summary of Approach Selection

- **Posterior Approach**: Preferred for cases needing extensive stabilization or when posterior elements are injured.
- **Anterior Approach**: Chosen for direct decompression in cases with significant anterior compression and single-level injury.
- **Combined Approach**: Recommended for complex injuries that demand both decompression and extensive stabilization.
- **Minimally Invasive Surgery**: Suitable for less severe or stable fractures where reduced muscle disruption and quicker recovery are beneficial.

The choice of surgical approach should be tailored to the individual patient, balancing the need for decompression, stabilization, and overall recovery goals.

11. When is laminectomy necessary for patients with acute thoracolumbar spinal cord injury?	Laminectomy, a surgical procedure involving the removal of part of the vertebra (the lamina) to decompress the spinal canal, is sometimes necessary in patients with acute thoracolumbar spinal cord injury (SCI). However, its use in thoracolumbar injuries is specific and less common than in cervical injuries due to the unique structural and neurological considerations in the thoracolumbar region. Here are the primary indications for laminectomy in these cases:<br>**1. Spinal Cord or Cauda Equina Compression**<br>• **Direct Neural Compression**: Laminectomy is indicated if there is direct compression on the spinal cord or cauda equina due to bone fragments, hematoma, or soft tissue impingement.<br>• **Incomplete SCI with Compressive Lesion**: Patients with incomplete SCI who have neurological deficits due to localized compression at the thoracolumbar junction (T10–L2) may benefit from decompression via laminectomy to relieve pressure on neural elements.<br>**2. Burst Fractures with Retropulsed Bone Fragments**<br>• **Significant Canal Compromise**: Laminectomy may be used when burst fractures cause vertebral body fragments to retropulse into the spinal canal, leading to compression of the spinal cord or cauda equina.<br>• **Inaccessible Fragments**: In cases where retropulsed fragments cannot be safely removed via an anterior approach, a posterior laminectomy can be used as an adjunct for indirect decompression.<br>**3. Severe Spinal Stenosis Post-Injury**<br>• **Preexisting Spinal Stenosis**: Patients with preexisting stenosis (narrowing of the spinal canal) who suffer an acute injury may need a laminectomy to create adequate space for the spinal cord or cauda equina.<br>• **Post-Traumatic Stenosis**: Laminectomy may also be considered in cases where traumatic changes exacerbate stenosis and contribute to neural compression.<br>**4. Posterior Ligamentous Complex Injury with Compression**<br>• **Associated with Ligamentous Disruption**: In injuries where the posterior ligamentous complex is disrupted and there is significant posterior element instability or compression, laminectomy may be part of a posterior approach to decompress and stabilize the spine.<br>• **Severe Kyphotic Deformity**: When kyphotic deformity resulting from posterior instability is causing cord compression, laminectomy can be combined with stabilization to restore alignment.

	**5. Laminectomy as Part of a Combined Approach**<br>• **Complementary to Anterior Decompression**: In complex injuries that require both anterior and posterior approaches, a laminectomy might be performed along with anterior decompression for optimal canal clearance.<br>• **Facilitate Instrumentation**: In certain cases, laminectomy may be done to allow for posterior instrumentation, especially when the laminae are damaged or obstructing the placement of pedicle screws.<br>**6. Progressive Neurological Deficit**<br>• **Worsening Neurological Function**: In patients with progressive neurological deficits despite conservative treatment or posterior stabilization, a laminectomy may be considered as an additional decompression strategy.<br>**Considerations and Limitations of Laminectomy in Thoracolumbar SCI**<br>• **Stabilization Needs**: Laminectomy alone may destabilize the spine further, especially in injuries involving burst fractures or ligamentous disruption. In most cases, it is combined with posterior instrumentation (e.g., screws and rods) to ensure stability.<br>• **Thoracolumbar Junction**: At the thoracolumbar junction (T10–L2), the spinal cord transitions to the cauda equina, and injuries here may involve both elements. Laminectomy must be carefully planned to address the specific type of neural compression.<br>• **Indications for Avoidance**: If there is no significant canal compromise or neural compression, laminectomy is generally avoided to preserve the integrity of the posterior elements.<br>**Summary**<br>Laminectomy is indicated in cases of acute thoracolumbar SCI where there is substantial spinal canal compromise, particularly from retropulsed fragments or severe stenosis contributing to neural compression. It is most effective when combined with posterior stabilization techniques to maintain spinal alignment and prevent further injury.
12. How to select the fixation segment for posterior surgery in patients with acute thoracolumbar spinal cord injury?	Selecting the appropriate fixation segment in posterior surgery for patients with acute thoracolumbar spinal cord injury (SCI) is crucial to achieving spinal stability, alignment, and effective healing. Here are the main principles and considerations for determining the fixation segment:<br>**1. Fracture Level and Type of Injury**<br>• **Single-Level Injury**: For isolated fractures with minimal instability, fixation typically spans one level above and one level below the fracture.

	• **Multilevel or Complex Injuries**: In cases with multilevel fractures or complex fracture-dislocation injuries, fixation may need to extend two levels above and two levels below the injury site.<br>• **Type of Fracture**:<br>○ **Compression Fractures**: Often stable, usually requiring a shorter fixation segment.<br>○ **Burst or Chance Fractures**: Typically need stabilization across multiple levels due to instability.<br>○ **Fracture-Dislocation**: Requires more extensive stabilization, often at least two levels above and below the fracture due to severe instability.<br>**2. Posterior Ligamentous Complex (PLC) Integrity**<br>• **Intact PLC**: If the posterior ligamentous complex is intact, shorter fixation segments (one level above and below) may suffice.<br>• **Disrupted PLC**: For injuries with significant ligamentous disruption, longer fixation is typically necessary to provide adequate stabilization, as the injury is highly unstable.<br>**3. Spinal Segment Characteristics (Transition Zones)**<br>• **Thoracolumbar Junction (T10–L2)**: This is a transition zone between the rigid thoracic and more flexible lumbar spine, which experiences higher biomechanical stresses. Injuries here often require extended fixation (two levels above and below) to ensure stability.<br>• **Junctional Regions**: If the injury is near a junctional area (e.g., thoracolumbar or lumbosacral junction), consider extending the fixation to adjacent stable segments to reduce the risk of junctional failure.<br>**4. Bone Quality**<br>• **Poor Bone Quality (e.g., Osteoporosis)**: In patients with osteoporosis or poor bone quality, longer fixation segments may be necessary to distribute forces and reduce the risk of hardware loosening or failure.<br>• **Normal Bone Quality**: In younger patients with good bone quality, shorter fixation segments may be sufficient, provided the injury is not highly unstable.<br>**5. Neurological Considerations**<br>• **Neurological Deficit**: In patients with neurological deficits due to retropulsed fragments, fixation may be extended to provide additional stability for decompression and to prevent further neurological compromise.<br>• **No Neurological Deficit**: In stable injuries without neurological compromise, the fixation segment can often be shorter,

	particularly if the fracture is localized and ligamentous structures are intact.<br>**6. Alignment Goals and Reduction Needs**<br>• **Significant Deformity**: In cases with kyphotic deformity or vertebral collapse, longer fixation may be necessary to restore and maintain proper spinal alignment.<br>• **Reduction and Stability**: For fractures requiring reduction (e.g., burst fractures with vertebral body collapse), extended fixation segments provide greater stability to maintain alignment post-reduction.<br>**7. Fixation Principles**<br>• **Pedicle Screw Fixation**: Pedicle screws are placed in vertebrae above and below the fracture level to achieve stabilization. The number of levels depends on the factors listed above.<br>• **Cross-Linking and Rods**: Cross-links and rods can enhance stability, especially in cases with extensive ligamentous disruption or rotational instability.<br>• **Short vs. Long Segment Fixation**:<br>○ **Short Segment**: Fixation of one level above and below, typically suitable for stable fractures with intact ligaments and no severe deformity.<br>○ **Long Segment**: Fixation extending two or more levels above and below, often necessary for complex, unstable injuries with significant ligamentous disruption or deformity.<br>**8. Minimizing Junctional Stress**<br>• **Avoid Fixation Termination at Junctional Levels**: Avoid ending the fixation at transition zones (e.g., T10-L2 or L5-S1), as these are high-stress areas. Instead, terminate fixation on stable vertebrae above or below these regions.<br>• **Extended Instrumentation**: If the injury is close to a junctional zone, extend the instrumentation across the junctional zone to prevent junctional kyphosis or hardware failure.<br>**Summary of Fixation Segment Selection**<br>• **Short Segment Fixation**: Generally recommended for single-level, stable fractures with intact ligamentous structures.<br>• **Long Segment Fixation**: Indicated for multilevel, highly unstable fractures, fractures near junctional areas, or cases with poor bone quality.<br>• **Special Considerations**: Transition zones, neurological deficit, ligamentous injury, bone quality, and alignment goals should

	all influence the final decision.<br>The goal of fixation is to provide stability for healing, maintain alignment, and protect neurological function. Decisions should be tailored to the specific injury and patient factors to achieve optimal surgical outcomes.
13. Is it necessary to fix the injured vertebrae posteriorly in patients with acute thoracolumbar spinal cord injury?	In patients with acute thoracolumbar spinal cord injury (SCI), it is not always necessary to fix the injured vertebra itself during posterior stabilization. Whether or not to include the injured vertebra in the fixation depends on several factors, including the type and severity of the fracture, stability, and the goals of surgery. Here's a breakdown of considerations:<br>**1. Type of Fracture**<br>• **Stable Compression Fractures**: In stable compression fractures without significant vertebral body collapse or instability, fixation can often be limited to adjacent vertebrae (one level above and below the injury), without including the fractured vertebra itself.<br>• **Unstable Burst or Fracture-Dislocation Injuries**: In highly unstable fractures, such as burst fractures with retropulsed fragments or fracture-dislocations, it may be beneficial to fix the injured vertebra to enhance stability, especially if there is significant displacement or risk of further collapse.<br>**2. Posterior Ligamentous Complex (PLC) Integrity**<br>• **Intact PLC**: If the posterior ligamentous complex is intact, the injured vertebra may not need to be directly fixed, as the adjacent levels can provide sufficient stability.<br>• **Disrupted PLC**: When the PLC is disrupted, including the injured vertebra in the fixation construct provides additional stability to the injured segment, helping prevent further deformity or collapse.<br>**3. Bone Quality and Risk of Collapse**<br>• **Poor Bone Quality (e.g., Osteoporosis)**: Patients with poor bone quality are at higher risk for progressive collapse of the injured vertebra. Fixing the injured vertebra can help maintain alignment and reduce the risk of kyphotic deformity.<br>• **Good Bone Quality**: In younger patients or those with good bone quality, the injured vertebra may not need direct fixation if the fracture is stable and there is no risk of further collapse.<br>**4. Extent of Vertebral Body Injury**<br>• **Minimal Vertebral Collapse**: When there is minimal collapse or height loss in the injured vertebra, fixation can often exclude the vertebra itself.

	• **Significant Vertebral Body Collapse**: If the injured vertebra has sustained significant collapse, including it in the fixation can help restore alignment and maintain stability, especially in cases requiring reduction of kyphotic deformity.<br>**5. Fixation Strategy (Short vs. Long Segment)**<br>• **Short-Segment Fixation**: In cases where only one level above and below is being fixed (short-segment fixation), including the injured vertebra provides added stability to a less extensive construct. This is common in fractures with moderate instability.<br>• **Long-Segment Fixation**: For long-segment constructs (two or more levels above and below), fixation may sometimes exclude the injured vertebra if it is not severely compromised, as the extended construct itself offers substantial stability.<br>**6. Biomechanical Considerations**<br>• **Preventing Postoperative Kyphosis**: Including the injured vertebra in the fixation helps to prevent postoperative kyphosis, especially in fractures with high risk of vertebral collapse or deformity.<br>• **Stress Distribution**: Fixing the injured vertebra helps distribute mechanical forces more evenly across the construct, which may reduce stress on the hardware and prevent screw loosening or breakage, particularly in unstable fractures.<br>**7. Neurological Considerations**<br>• **Spinal Canal Compromise**: In cases where the injured vertebra has retropulsed fragments causing canal compromise, including it in the fixation allows for stabilization during decompression procedures.<br>• **No Canal Compromise**: If there is no canal compromise or spinal cord involvement, fixation can sometimes exclude the injured vertebra if the fracture is stable.<br>**Summary**<br>Including the injured vertebra in the posterior fixation construct can enhance stability, prevent kyphotic deformity, and improve outcomes in unstable injuries or cases with poor bone quality. However, in stable fractures with minimal vertebral collapse and intact ligamentous structures, excluding the injured vertebra may be feasible. The decision should be individualized based on fracture characteristics, patient factors, and surgical goals to optimize spinal stability and alignment.
14. What are the indications for percutaneous internal fixation in patients with acute thoracolumbar spinal cord injury?	Percutaneous internal fixation is a minimally invasive surgical technique used in selected patients with acute thoracolumbar spinal cord injury (SCI) to stabilize the spine while reducing soft tissue disruption. This approach is particularly beneficial for certain fracture types and patient profiles. Here are the primary indications for percutaneous internal fixation in these patients:

1. Stable and Unstable Fractures with Minimal Neural Compression

- **Compression and Burst Fractures**: Patients with compression or burst fractures, especially those with minimal or no spinal canal compromise, are good candidates for percutaneous internal fixation.
- **No Severe Retropulsed Fragments**: When there is no significant retropulsion of bone fragments into the spinal canal causing spinal cord or cauda equina compression, percutaneous fixation is feasible.

2. Intact or Minimally Disrupted Posterior Ligamentous Complex (PLC)

- **Stable Posterior Structures**: Percutaneous fixation is ideal when the posterior ligamentous complex is either intact or only minimally compromised. This ensures stability can be maintained without the need for extensive open decompression.
- **Low-Risk Instability**: For fractures where the posterior ligamentous complex is disrupted, percutaneous fixation may still be used as a temporary measure, particularly in polytrauma patients who may require staged open procedures later.

3. Polytrauma Patients with Multiple Injuries

- **High-Risk Patients**: For patients with multiple traumatic injuries, percutaneous internal fixation can provide rapid spinal stabilization while minimizing operative time and blood loss.
- **Temporary Stabilization**: In polytrauma cases, it can serve as a temporary stabilization technique, allowing the patient to undergo treatment for other critical injuries before potentially receiving a definitive spinal procedure.

4. Patients with Medical Comorbidities

- **High Surgical Risk**: Patients with significant comorbidities (e.g., cardiopulmonary disease, obesity) who may not tolerate extensive open surgery may benefit from the less invasive nature of percutaneous fixation.
- **Reduced Blood Loss and Operative Time**: Minimally invasive techniques reduce blood loss and operative time, which can be advantageous for patients with limited physiological reserve.

5. Need for Short-Segment Fixation

- **Limited to One or Two Levels Above and Below**: Percutaneous fixation is most effective in cases where short-segment stabilization (one or two levels above and below the fracture) is sufficient.
- **Localized Fractures**: For single-level fractures or injuries with minimal displacement, short-segment percutaneous fixation is often sufficient to provide stability without the need for long-segment constructs.

6. Minimizing Soft Tissue Disruption and Postoperative Pain

- **Cosmetic and Functional Benefits**: Patients who may benefit from quicker recovery and reduced postoperative pain, such as younger, active individuals, are often suitable candidates for percutaneous fixation.
- **Avoiding Extensive Muscle Dissection**: By minimizing soft tissue dissection, percutaneous fixation reduces muscle damage, which can lower the risk of postoperative pain and complications.

7. Patients with Osteoporotic or Fragile Bone Quality

- **Less Invasive Option for Fragile Bones**: Patients with osteoporosis or poor bone quality can benefit from percutaneous fixation, as it places less stress on fragile bones compared to open fixation.
- **Risk of Screw Loosening**: In some cases, percutaneous techniques may reduce the risk of screw loosening by using shorter and strategically placed screws, especially when combined with cement augmentation techniques.

8. Absence of Severe Kyphotic Deformity

- **Mild to Moderate Deformity**: Patients with fractures that have caused minimal kyphotic deformity or vertebral collapse are better suited for percutaneous fixation. Severe deformities often require open surgery to allow for realignment and deformity correction.
- **Deformity Prevention**: Percutaneous fixation can help stabilize the spine early on, preventing further deformity in mildly displaced fractures.

Summary of Indications for Percutaneous Internal Fixation

- **Fracture Type**: Compression or burst fractures with minimal spinal canal compromise.
- **Posterior Ligament Integrity**: Intact or minimally disrupted posterior ligamentous complex.
- **Polytrauma Patients**: Temporary or definitive stabilization in patients with other critical injuries.
- **Comorbidities**: Suitable for patients with medical comorbidities who are at high surgical risk.
- **Short-Segment Fixation**: Appropriate for localized fractures that don't require extensive fixation.
- **Osteoporotic Patients**: Useful in patients with fragile bone quality when combined with augmentation techniques.
- **Minimal Deformity**: Effective in patients with limited vertebral collapse and no severe kyphosis.

Percutaneous internal fixation is an effective, less invasive option for stabilizing specific types of thoracolumbar injuries, particularly when minimizing operative trauma is a priority. However, it is generally not recommended for cases with severe canal compromise, high degrees of instability, or significant kyphotic deformity that require open reduction and more extensive stabilization.

15. Is it necessary to perform bone graft fusion for patients with acute thoracolumbar spinal cord injury who undergo surgery?	Bone graft fusion is not always necessary for all patients with acute thoracolumbar spinal cord injury (SCI) undergoing surgery. The decision to perform fusion depends on the type of fracture, the stability of the injury, and the surgical goals. Here's an overview of when bone graft fusion is generally indicated and when it may not be necessary:<br>**1. Indications for Bone Graft Fusion**<br>• **Unstable Fractures with Posterior Ligamentous Complex (PLC) Disruption**<br>○ In cases where the PLC is disrupted (e.g., fracture-dislocations, burst fractures with ligamentous injury), fusion is often recommended to achieve long-term stability.<br>○ Disruption of the PLC compromises spinal stability, and fusion helps restore structural integrity and prevent future instability or deformity.<br>• **Severe Burst Fractures with Vertebral Body Collapse**<br>○ When there is significant vertebral body collapse, particularly in burst fractures, bone graft fusion provides additional structural support.<br>○ Fusion in these cases prevents progressive kyphotic deformity, which can result from the weakened vertebral body.<br>• **Multilevel or Complex Injuries Requiring Extensive Stabilization**<br>○ For fractures involving multiple vertebrae or complex injury patterns that require long-segment stabilization, fusion is often necessary.<br>○ Fusion across multiple levels ensures that the fixation construct remains stable, reducing the risk of hardware failure.<br>• **Patients with Poor Bone Quality (e.g., Osteoporosis)**<br>○ For patients with osteoporosis or other conditions that weaken bone, fusion with bone grafting provides additional support and promotes bone healing, helping prevent future vertebral collapse.<br>○ Fusion may reduce the risk of screw loosening and hardware failure in patients with fragile bone.<br>• **Fractures with Significant Deformity or Malalignment**<br>○ In cases with preexisting or injury-induced spinal deformity (e.g., kyphosis), fusion helps maintain alignment after surgical correction.

	- The fusion prevents the deformity from progressing over time, especially in areas of high biomechanical stress like the thoracolumbar junction.<br>**2. When Bone Graft Fusion May Not Be Necessary**<br>• **Stable Fractures with Intact PLC**<br>- In compression fractures or stable burst fractures where the PLC is intact, fusion may not be necessary.<br>- For these injuries, short-segment fixation may provide sufficient stability without the need for fusion, allowing for possible implant removal after healing.<br>• **Single-Level, Minimally Displaced Fractures**<br>- In single-level fractures with minimal displacement and no risk of progressive deformity, fusion is often not required.<br>- Short-segment fixation alone may suffice, especially if the vertebral body is mostly intact and the injury does not impact spinal alignment.<br>• **Younger Patients with Good Healing Potential**<br>- In young, healthy patients with good bone quality, fixation alone may be sufficient in certain stable fractures, as these patients have a higher potential for bone healing.<br>- Avoiding fusion can help preserve spinal mobility, especially in the thoracolumbar junction where flexibility is valuable.<br>• **Temporary Stabilization in Polytrauma Patients**<br>- In polytrauma patients where temporary stabilization is needed, fusion may not be performed initially.<br>- These patients may undergo temporary percutaneous fixation without fusion, allowing for later reassessment once their overall condition stabilizes.<br>**3. Benefits of Bone Graft Fusion**<br>• **Long-Term Stability**: Fusion provides long-term stability in unstable fractures, preventing progressive deformity and reducing the risk of hardware failure.<br>• **Deformity Prevention**: Fusion helps maintain proper spinal alignment, especially in cases with kyphotic deformity or vertebral body collapse.

	• **Reduced Risk of Reoperation**: By fusing the unstable segment, the risk of requiring future surgery for instability is reduced.<br>**4. Risks and Considerations of Bone Graft Fusion**<br>• **Loss of Spinal Mobility**: Fusion reduces mobility at the fused segments, which may impact function, especially if multiple levels are fused.<br>• **Increased Surgical Complexity**: Fusion procedures are more complex and may require additional operative time, increased blood loss, and longer recovery.<br>• **Potential for Adjacent Segment Degeneration**: Fusion increases stress on adjacent levels, which may lead to degeneration over time, particularly in the thoracolumbar region.<br>**Summary**<br>Bone graft fusion is typically necessary for patients with unstable or complex thoracolumbar fractures, disrupted posterior ligamentous structures, or those at risk of progressive deformity. For stable, single-level fractures with minimal displacement and intact ligaments, fusion may not be required, especially in younger patients with good bone quality. The decision to perform fusion should be individualized, balancing the need for stability with the goal of preserving as much spinal function and mobility as possible.
16. Is simple pedicle-based bone grafting effective in patients with acute thoracolumbar spinal cord injury?	Simple pedicle-based bone grafting alone is generally not considered effective for most patients with acute thoracolumbar spinal cord injury (SCI). While it may offer limited support in specific, stable cases, its ability to provide sufficient stability is often inadequate due to the high biomechanical demands of the thoracolumbar region. Here's an analysis of why simple pedicle-based bone grafting may have limited effectiveness and in what cases it might be considered:<br>**1. Limited Stability for High-Stress Areas**<br>• **Biomechanical Demands**: The thoracolumbar junction (T10–L2) is a transition zone between the relatively rigid thoracic spine and the more mobile lumbar spine, and it is subject to high biomechanical stress. Simple pedicle-based bone grafting often cannot provide enough support to stabilize this area adequately.<br>• **Risk of Collapse or Malalignment**: Without additional stabilization, there is a high risk of graft subsidence (sinking), vertebral collapse, or progressive kyphotic deformity, especially in cases with even mild instability.<br>**2. Effective Only for Highly Stable, Minimal Injuries**<br>• **Stable Compression Fractures**: Simple pedicle-based bone grafting may offer some support in highly stable, minimal compression fractures where the posterior ligamentous complex (PLC) is intact and there is no vertebral displacement or

significant deformity.

- **Minimal Displacement and Intact Ligaments**: It might be considered in cases where there is no significant vertebral body collapse, displacement, or PLC injury, as the stability requirements in such cases are lower.

3. Inadequate for Unstable or Complex Fractures

- **Unstable Fractures**: In cases of unstable fractures, burst fractures, or fracture-dislocations, simple pedicle-based grafting alone does not provide adequate stability. These types of injuries generally require additional fixation (e.g., pedicle screws with rods) to maintain alignment and stability.
- **Ligamentous Complex Injury**: If the PLC or other supportive structures are compromised, simple pedicle-based grafting will not restore stability, and such injuries often require more extensive stabilization with bilateral or multi-level fixation.

4. Limited Utility in Preventing Deformity

- **Kyphotic Deformity Risk**: Simple grafting does not provide the necessary support to prevent kyphotic deformity in cases where there is a risk of vertebral body collapse or malalignment. Long-term outcomes may include progressive deformity, which can lead to chronic pain or neurological compromise.
- **Inadequate for Alignment Correction**: In cases where alignment correction is needed (e.g., post-traumatic kyphosis), simple pedicle-based grafting alone is insufficient without additional structural support from other fixation techniques.

5. Potential Role as an Auxiliary Technique

- **Combination with Posterior Fixation**: In select, stable cases, simple pedicle-based bone grafting could be used as an adjunct to posterior fixation, especially in patients with good bone quality and minimal instability. However, it is generally not sufficient as a standalone treatment.
- **Minimally Invasive Considerations**: For patients requiring a minimally invasive approach with very stable fractures, it may be considered as part of a conservative treatment approach, but its application is limited.

Summary

Overall, simple pedicle-based bone grafting is not typically effective as a standalone treatment for most patients with acute thoracolumbar SCI. It may provide limited benefits in highly stable, minor injuries with intact ligamentous support and no significant displacement. However, in most cases, especially those with moderate to severe instability, additional stabilization (e.g., bilateral pedicle screws, rods, or interbody fusion) is necessary to ensure proper alignment, stability, and prevention of deformity.

17. How to manage the urinary system in patients with acute thoracolumbar spinal cord injury?	Managing the urinary system in patients with acute thoracolumbar spinal cord injury (SCI) is essential to prevent complications such as urinary retention, infections, and long-term dysfunction. Here are the key steps for managing the urinary system in these patients:<br>**1. Initial Assessment and Bladder Management**<br>● **Assess Bladder Function**: Evaluate the patient's level of injury and neurological status to determine potential bladder dysfunction. Thoracolumbar injuries can disrupt bladder function, leading to either neurogenic bladder or urinary retention.<br>● **Insert an Indwelling Catheter**: For immediate management, place a Foley catheter to prevent urinary retention and monitor urine output accurately. This also helps prevent overdistension of the bladder, which can lead to detrusor muscle damage.<br>**2. Catheterization Techniques**<br>● **Intermittent Catheterization**: After the initial acute phase, consider transitioning to intermittent catheterization to reduce infection risk. This involves catheterizing the bladder at regular intervals (every 4–6 hours) to empty it completely.<br>○ **Benefits**: Intermittent catheterization reduces the risk of urinary tract infections (UTIs) associated with indwelling catheters and helps maintain bladder capacity.<br>○ **Patient Education**: Teach the patient and caregivers proper catheterization techniques to ensure safe and effective bladder emptying.<br>● **Indwelling Catheter for Patients Unable to Self-Catheterize**: If intermittent catheterization is not feasible (due to physical limitations or caregiver support), a long-term indwelling catheter may be used temporarily, though it carries a higher risk of UTIs and other complications.<br>**3. Bladder Training and Monitoring**<br>● **Bladder Training Program**: Once the patient's condition stabilizes, initiate a bladder training program to encourage regular bladder emptying. This involves scheduled voiding attempts with or without catheterization, depending on residual bladder function.<br>● **Monitor Post-Void Residual (PVR)**: Regularly assess PVR volumes using ultrasound or catheterization to ensure the bladder is emptying adequately and to determine whether adjustments to the bladder management plan are necessary.<br>● **Fluid Management**: Encourage adequate hydration to maintain healthy urine output and reduce the risk of UTIs. However, avoid excessive fluid intake, which can increase bladder pressures.<br>**4. Medications to Assist Bladder Function**

- **Anticholinergic Medications**: Prescribe anticholinergics (e.g., oxybutynin, tolterodine) if the patient experiences bladder spasms or overactivity, which can cause incontinence or high bladder pressures.
- **Alpha-Blockers**: In some cases, alpha-blockers (e.g., tamsulosin) can help relax the bladder neck and improve urine flow, reducing the risk of urinary retention.
- **Botulinum Toxin (Botox) Injections**: In patients with severe detrusor overactivity unresponsive to oral medications, Botox injections into the bladder wall can help relax the bladder and prevent high pressures.

5. Preventing and Managing Urinary Tract Infections (UTIs)

- **Aseptic Catheterization Techniques**: Whether using intermittent or indwelling catheterization, ensure that aseptic techniques are followed to reduce the risk of introducing bacteria into the urinary tract.
- **Monitor for Signs of Infection**: Regularly check for signs of UTIs (e.g., fever, cloudy urine, discomfort) and treat promptly with antibiotics if infections occur.
- **Prophylactic Antibiotics**: Long-term antibiotic prophylaxis is generally avoided due to the risk of antibiotic resistance, but short-term prophylaxis may be considered in patients with recurrent UTIs.

6. Long-Term Bladder Management

- **Urodynamic Studies**: After the acute phase and once the patient's condition has stabilized, consider conducting urodynamic studies to assess bladder function and determine an appropriate long-term management plan.
- **Continence and Quality of Life Goals**: Discuss bladder management goals with the patient, aiming for a program that balances continence, bladder health, and quality of life.
- **Surgical Options**: In chronic cases where conservative management is ineffective, surgical options like bladder augmentation, sphincterotomy, or creation of a suprapubic catheter may be considered.

7. Education and Support

- **Patient and Caregiver Education**: Educate the patient and caregivers on bladder management techniques, signs of complications, and the importance of regular follow-up to ensure effective long-term care.
- **Self-Catheterization Training**: If feasible, train the patient to perform self-catheterization, as this provides more independence and control over bladder management.

Summary

	Managing the urinary system in patients with acute thoracolumbar SCI requires a structured approach to prevent complications, maintain bladder health, and optimize quality of life. Early catheterization, regular monitoring, and bladder training are essential steps, along with medication and potentially surgical interventions for long-term management. Education and support for patients and caregivers are crucial for successful bladder management and prevention of secondary complications.
18. How to prevent and treat deep vein thrombosis in patients with acute thoracolumbar spinal cord injury?	Preventing and treating deep vein thrombosis (DVT) in patients with acute thoracolumbar spinal cord injury (SCI) is crucial, as these patients are at high risk for venous thromboembolism due to immobility and impaired venous return. Here are key strategies for prevention and treatment:<br>**1. Early Risk Assessment**<br>• **Assess DVT Risk**: Evaluate all SCI patients for DVT risk upon admission, considering factors such as immobility, age, obesity, history of DVT, and any known coagulopathies.<br>• **Individualized Plan**: Develop an individualized DVT prophylaxis plan based on the patient's risk factors, neurological level of injury, and contraindications for certain interventions.<br>**2. Mechanical Prophylaxis**<br>• **Intermittent Pneumatic Compression (IPC) Devices**: IPC devices, which periodically inflate around the legs, are recommended to improve venous return and reduce stasis. They should be applied as early as possible and used consistently, especially if pharmacologic prophylaxis is delayed.<br>• **Graduated Compression Stockings**: Compression stockings (e.g., 18–20 mmHg) can also be used to maintain venous return in the lower limbs. Ensure proper fitting to avoid discomfort or skin breakdown.<br>• **Passive Range of Motion Exercises**: If feasible, implement passive range of motion exercises for the lower limbs to stimulate circulation, ideally under the supervision of a physical therapist.<br>**3. Pharmacologic Prophylaxis**<br>• **Low Molecular Weight Heparin (LMWH)**: LMWH, such as enoxaparin, is commonly used for DVT prophylaxis in SCI patients due to its effectiveness and ease of administration. Begin LMWH within 24–72 hours of injury if there are no contraindications (e.g., active bleeding or planned surgery).<br>• **Unfractionated Heparin (UFH)**: If LMWH is contraindicated or unavailable, UFH may be used, especially in patients requiring more frequent monitoring.

- **Duration of Prophylaxis**: Continue pharmacologic prophylaxis for at least 8–12 weeks or until the patient is mobilized, as SCI patients have prolonged risk for DVT.

4. Monitor for Signs of DVT and Pulmonary Embolism (PE)

- **Regular Physical Examination**: Monitor for symptoms of DVT, such as unilateral leg swelling, warmth, tenderness, or redness, as well as signs of PE, like shortness of breath, chest pain, or sudden hypotension.
- **Routine Ultrasound Screening**: Consider periodic Doppler ultrasound screening of the lower extremities, particularly if DVT risk is high or if symptoms develop.

5. Treatment of DVT

- **Initiate Anticoagulation**: If DVT is diagnosed, start anticoagulation therapy immediately, typically with LMWH or a direct oral anticoagulant (DOAC), unless contraindicated.
- **Transition to Oral Anticoagulants**: After the initial phase with LMWH or UFH, patients may be transitioned to a long-term oral anticoagulant, such as warfarin or a DOAC, for 3–6 months depending on the severity of the DVT and risk of recurrence.
- **Inferior Vena Cava (IVC) Filter**: If anticoagulation is contraindicated (e.g., due to active bleeding or recent surgery), consider an IVC filter to prevent pulmonary embolism. This is typically a temporary solution until anticoagulation can be safely resumed.

6. Physical Therapy and Early Mobilization

- **Encourage Early Mobilization**: Begin mobilization as soon as medically feasible, with support from a physical therapist. Even sitting up or limited ambulation can help improve circulation and reduce DVT risk.
- **Progressive Mobilization**: Gradually progress mobility activities as tolerated, following a structured physical therapy plan to prevent venous stasis.

7. Monitoring and Adjusting Prophylaxis

- **Regular Monitoring of Anticoagulation**: For patients on heparin or warfarin, regularly monitor coagulation markers (e.g., INR for warfarin) to ensure therapeutic levels are maintained without causing excessive bleeding.
- **Adjust Prophylaxis Post-Surgery**: If surgery is required, hold anticoagulation for the appropriate period before and after surgery to reduce bleeding risks, then resume prophylaxis as soon as possible.

8. Patient Education and Long-Term Management

	• **Educate Patients and Caregivers**: Explain the importance of DVT prevention measures, including compliance with mechanical devices and symptoms to watch for, such as leg pain or shortness of breath.<br>• **Long-Term Follow-Up**: SCI patients remain at higher risk for DVT, so continue DVT prevention strategies even after discharge if risk factors persist. Regular follow-up appointments should include monitoring for signs of DVT or PE recurrence.<br>**Summary**<br>To prevent and treat DVT in acute thoracolumbar SCI patients, implement a comprehensive plan involving early risk assessment, consistent mechanical and pharmacologic prophylaxis, regular monitoring, and timely intervention for diagnosed DVT. Early mobilization and patient education are also essential for long-term prevention and overall recovery.
19. Is it necessary to prevent and treat pressure sores in patients with acute thoracolumbar spinal cord injury?	Yes, it is essential to prevent and treat pressure sores in patients with acute thoracolumbar spinal cord injury (SCI). These patients are at high risk for developing pressure sores due to immobility, sensory deficits, and impaired blood flow, which can lead to skin breakdown, infection, and other serious complications. Here's a guide to why it's necessary and how to manage pressure sore prevention and treatment in these patients:<br>**1. Importance of Preventing Pressure Sores**<br>• **High Risk Due to Immobility**: Patients with SCI often experience limited mobility, reducing their ability to shift positions and relieve pressure on vulnerable skin areas.<br>• **Sensory Deficits**: SCI can result in loss of sensation below the injury level, preventing patients from feeling discomfort or early signs of skin breakdown.<br>• **Complications**: Untreated pressure sores can lead to infection, sepsis, delayed rehabilitation, and prolonged hospital stays, significantly affecting patient outcomes and quality of life.<br>**2. Prevention Strategies**<br>• **Regular Repositioning**: Position changes should be done every 2 hours if the patient is bedridden, or every 15–30 minutes if the patient is in a wheelchair. This relieves pressure on vulnerable areas like the sacrum, buttocks, heels, and elbows.<br>• **Pressure-Relieving Devices**:<br>○ **Specialized Mattresses and Cushions**: Use pressure-relieving mattresses (e.g., foam, air, or gel overlays) and wheelchair cushions to evenly distribute body weight and reduce pressure on high-risk areas.<br>○ **Heel Protectors**: Soft heel protectors or cushions can prevent pressure sores on the heels, which are particularly

	prone to breakdown.<br>• **Skin Care and Inspection**:<br>○ **Daily Skin Checks**: Inspect the skin at least once a day for signs of redness, warmth, or breakdown, especially on bony prominences.<br>○ **Keep Skin Clean and Dry**: Gently cleanse the skin with mild soap and water, ensuring thorough drying, particularly in moist areas (e.g., groin and buttocks).<br>○ **Moisturize**: Use moisturizers to prevent dryness, but avoid excessive moisture, as it can soften the skin and increase the risk of breakdown.<br>• **Nutrition and Hydration**:<br>○ **Adequate Protein Intake**: A diet high in protein supports skin health and healing. Vitamins and minerals like zinc and vitamin C also contribute to skin integrity.<br>○ **Maintain Hydration**: Adequate hydration helps keep the skin resilient and reduces the risk of breakdown.<br>**3. Education and Patient Involvement**<br>• **Patient and Caregiver Education**: Educate patients and caregivers on the importance of pressure sore prevention, skin inspection, and position changes.<br>• **Encourage Self-Checking (If Possible)**: For patients with partial sensation or limited movement, encourage self-checking and awareness of pressure areas when possible.<br>**4. Treatment of Existing Pressure Sores**<br>• **Early Intervention**: Begin treatment at the earliest sign of pressure sore development (e.g., redness or skin breakdown) to prevent progression to more severe stages.<br>• **Wound Care**:<br>○ **Stage I and II Pressure Sores**: Clean the wound gently, apply a protective dressing (e.g., hydrocolloid or foam), and reduce pressure on the area. For Stage II sores, dressings that maintain a moist environment can promote healing.<br>○ **Stage III and IV Pressure Sores**: These require more advanced wound care, often with debridement to remove dead tissue and the use of specialized dressings. In severe cases, surgical intervention may be necessary.

	• **Debridement**: For sores with necrotic tissue, debridement (removal of dead tissue) is essential to promote healing. This may involve enzymatic, autolytic, or surgical methods based on the severity and condition of the wound.<br>• **Infection Management**: Monitor for signs of infection (e.g., redness, swelling, foul odor, discharge) and treat with antibiotics if infection is confirmed. Infected pressure sores can quickly worsen and lead to systemic complications.<br>• **Adjunct Therapies**: For non-healing or complex wounds, adjunct therapies like negative pressure wound therapy (NPWT) or electrical stimulation may be considered to promote wound healing.<br>**5. Long-Term Management and Monitoring**<br>• **Regular Follow-Up**: Continuous monitoring and regular follow-up are essential to ensure healing, prevent recurrence, and manage any complications.<br>• **Promote Functional Independence**: Teach patients ways to manage pressure relief on their own, if feasible, and involve physical and occupational therapists to support mobility and skin health.<br>**Summary**<br>Preventing and treating pressure sores in patients with acute thoracolumbar SCI is critical due to their high susceptibility and the potential for serious complications. A comprehensive approach combining regular repositioning, pressure-relieving devices, skin care, nutrition, and patient education can significantly reduce the risk of pressure sores. For existing sores, prompt wound care, debridement, infection control, and ongoing monitoring are essential to promote healing and prevent further deterioration.
20. How to manage neurogenic bowel in patients with acute thoracolumbar spinal cord injury?	Managing neurogenic bowel in patients with acute thoracolumbar spinal cord injury (SCI) is essential to maintain gastrointestinal function, prevent complications like constipation or incontinence, and enhance quality of life. Neurogenic bowel management requires a structured approach tailored to the patient's level of injury, bowel function, and overall health status. Here's a guide to managing neurogenic bowel in these patients:<br>**1. Assess Bowel Function and Set Goals**<br>• **Initial Assessment**: Evaluate the level of spinal injury and the type of neurogenic bowel dysfunction. Thoracolumbar injuries generally affect the function of the bowel by disrupting nerve signals, which can lead to reflex bowel (spastic) or areflexic bowel (flaccid), depending on the level and completeness of injury.<br>• **Establish Management Goals**: The goals are to maintain regular, complete bowel emptying, prevent constipation or impaction, avoid incontinence, and achieve a manageable routine for the patient.

2. Bowel Program and Routine

- **Establish a Regular Schedule**: A consistent bowel program, typically scheduled every 1–3 days, helps regulate bowel movements. Timing should consider the patient's usual bowel habits and lifestyle to promote adherence.
- **Timed Emptying**: Encourage emptying at the same time each day to take advantage of the body's natural reflexes, which are often strongest in the morning after a meal (gastrocolic reflex).

3. Dietary and Fluid Management

- **High-Fiber Diet**: Ensure a diet rich in fiber (e.g., fruits, vegetables, whole grains) to promote stool bulk and prevent constipation. Aim for 25–35 grams of fiber per day, adjusted based on tolerance and bowel response.
- **Adequate Hydration**: Adequate fluid intake (around 2–3 liters per day, unless contraindicated) helps soften stool and promotes regular bowel movements. Ensure hydration is balanced, as excessive fluid intake can increase bowel transit time, leading to loose stools.

4. Medications for Bowel Regulation

- **Stool Softeners**: Stool softeners, such as docusate sodium, can help soften stools and make bowel movements easier without causing dependency.
- **Fiber Supplements**: Fiber supplements (e.g., psyllium) may be beneficial if dietary fiber is insufficient, although they must be taken with adequate water to avoid impaction.
- **Stimulant Laxatives (as Needed)**: Use stimulant laxatives (e.g., senna or bisacodyl) sparingly to stimulate bowel contractions if constipation becomes an issue. Avoid frequent use to prevent dependence.
- **Suppositories or Enemas**: For patients with more severe neurogenic bowel dysfunction, rectal stimulants such as glycerin suppositories or mini-enemas can help trigger bowel movements as part of a scheduled bowel program.

5. Manual Techniques and Digital Stimulation

- **Digital Rectal Stimulation**: In reflex (spastic) bowel, digital rectal stimulation may help initiate bowel movements by stimulating rectal reflexes. This technique involves gentle circular motions with a lubricated gloved finger to trigger defecation reflexes.
- **Manual Evacuation**: For patients with areflexic (flaccid) bowel, manual evacuation of stool may be required if there is incomplete emptying. This should be done carefully and consistently to avoid trauma to the rectal mucosa.

6. Exercise and Physical Activity

- **Encourage Physical Movement**: Physical activity stimulates bowel motility, helping reduce constipation. Even gentle movement or passive exercises guided by a physical therapist can aid gastrointestinal function.
- **Abdominal Massage**: For some patients, gentle abdominal massage following the direction of the colon may promote bowel motility and help with stool movement through the intestines.

7. Preventing and Managing Complications

- **Constipation and Impaction**: Regularly monitor for signs of constipation or stool impaction, as these are common in SCI patients due to reduced motility. Adjust the bowel program and medications if constipation becomes problematic.
- **Incontinence**: For patients with reflex bowel and incontinence, managing the timing of bowel emptying can help minimize accidents. Scheduled bowel programs can reduce the likelihood of incontinence.
- **Hemorrhoids**: Frequent digital stimulation or manual evacuation can increase the risk of hemorrhoids, so use gentle techniques and ensure adequate lubrication. Treat hemorrhoids promptly to prevent pain and further complications.

8. Patient and Caregiver Education

- **Teach Techniques**: Educate the patient and caregivers on bowel management techniques, such as digital stimulation and use of medications, to empower them to manage the neurogenic bowel program.
- **Signs to Watch For**: Instruct patients and caregivers to recognize signs of complications, such as abdominal pain, hard stool, bloating, or incontinence, and when to seek medical attention.

9. Long-Term Monitoring and Adjustments

- **Regular Follow-Up**: Periodic follow-up with healthcare providers helps assess the effectiveness of the bowel program, adjust treatments as needed, and ensure the patient's comfort and quality of life.
- **Adjust Based on Response**: Over time, adjustments to the bowel program may be needed to adapt to changes in diet, medication, activity level, or overall health.

Summary

Managing neurogenic bowel in patients with acute thoracolumbar SCI involves creating a consistent bowel program that includes dietary adjustments, scheduled evacuations, medications as needed, and physical activity. Educating patients and caregivers is crucial to ensure adherence and effective self-management, while regular follow-up helps adjust the program for optimal outcomes. This

	structured approach improves bowel function, reduces complications, and enhances the patient's quality of life.<br>Yes, early surgical intervention can improve outcomes for patients with thoracic and lumbar fractures, especially when the fractures are unstable, involve neurological impairment, or pose a risk of further spinal cord or nerve injury. Here are some of the primary reasons why early surgical intervention can be beneficial:<br>**1. Neurological Recovery**<br>• **Decompression of Neural Elements**: In cases where fractures result in spinal cord or nerve root compression, early surgery allows for timely decompression, which may improve neurological recovery, particularly in incomplete injuries.<br>• **Prevention of Secondary Injury**: Early decompression helps reduce spinal cord edema, inflammation, and ischemia, which can worsen neurological deficits if left unaddressed.<br>**2. Spinal Stability and Alignment**<br>• **Stabilization of Unstable Fractures**: Early surgical fixation provides stability in unstable fractures, reducing the risk of further displacement or deformity that could compromise spinal cord or nerve function.<br>• **Alignment Correction**: Surgery can restore and maintain spinal alignment, preventing progressive deformities like kyphosis, which can lead to chronic pain, compromised lung function, or further neurological issues over time.<br>**3. Pain Reduction and Improved Mobility**<br>• **Reduction of Fracture-Related Pain**: Stabilizing the spine surgically can help reduce acute pain caused by the fracture, allowing patients to mobilize sooner with reduced discomfort.<br>• **Early Mobilization**: Surgical stabilization allows for early mobilization and rehabilitation, which can improve functional outcomes, reduce complications related to prolonged bed rest (e.g., deep vein thrombosis, pressure sores), and enhance recovery.<br>**4. Prevention of Complications Associated with Prolonged Immobilization**<br>• **Reduced Risk of Bed Rest Complications**: Early surgery allows patients to avoid the complications associated with prolonged immobilization, such as pneumonia, muscle atrophy, and pressure sores.<br>• **Improved Rehabilitation Potential**: Patients who are surgically stabilized can typically engage in physical therapy and rehabilitation sooner, which supports muscle strength, endurance, and overall recovery.<br>**5. Improved Long-Term Functional Outcomes**

	• **Enhanced Quality of Life**: Early surgical intervention has been shown to improve long-term outcomes by allowing patients to return to daily activities more quickly and with better functional capacity.<br>• **Lower Likelihood of Reoperation**: Early alignment and stabilization reduce the risk of progressive deformities and hardware failures that may necessitate additional surgery in the future.<br>**6. Evidence from Studies**<br>• Studies have shown that early surgical intervention (within 24–72 hours after injury) in patients with thoracic and lumbar fractures, particularly those with neurological deficits, is associated with better neurological recovery, decreased hospital stays, and improved overall outcomes compared to delayed surgery.<br>• The timing of surgery is most beneficial within the first 24–72 hours for neurological recovery, as this period allows for the best chance of mitigating secondary spinal cord injury from edema and inflammation.<br>**When Early Surgery May Not Be Indicated**<br>• **Medically Unstable Patients**: In patients who are medically unstable or have life-threatening injuries, stabilizing other critical conditions may take priority before spinal surgery.<br>• **Stable Fractures without Neurological Compromise**: For stable fractures without neurological involvement, non-surgical management may be effective, and early surgery may not significantly alter outcomes.<br>**Summary**<br>Early surgical intervention in patients with thoracic and lumbar fractures, particularly those with instability, neurological deficits, or risk of deformity, generally leads to better outcomes. By providing timely decompression, stabilization, and alignment correction, early surgery enhances neurological recovery, pain relief, functional mobility, and quality of life. Each case should be evaluated individually, considering the patient's overall health, fracture characteristics, and potential risks.
21.Does early surgical intervention improve outcomes for patients with thoracic and lumbar fractures?	Yes, early surgical intervention can improve outcomes for patients with thoracic and lumbar fractures, especially when the fractures are unstable, involve neurological impairment, or pose a risk of further spinal cord or nerve injury. Here are some of the primary reasons why early surgical intervention can be beneficial:<br>**1. Neurological Recovery**<br>• **Decompression of Neural Elements**: In cases where fractures result in spinal cord or nerve root compression, early surgery allows for timely decompression, which may improve neurological recovery, particularly in incomplete injuries.

- **Prevention of Secondary Injury**: Early decompression helps reduce spinal cord edema, inflammation, and ischemia, which can worsen neurological deficits if left unaddressed.

2. Spinal Stability and Alignment

- **Stabilization of Unstable Fractures**: Early surgical fixation provides stability in unstable fractures, reducing the risk of further displacement or deformity that could compromise spinal cord or nerve function.
- **Alignment Correction**: Surgery can restore and maintain spinal alignment, preventing progressive deformities like kyphosis, which can lead to chronic pain, compromised lung function, or further neurological issues over time.

3. Pain Reduction and Improved Mobility

- **Reduction of Fracture-Related Pain**: Stabilizing the spine surgically can help reduce acute pain caused by the fracture, allowing patients to mobilize sooner with reduced discomfort.
- **Early Mobilization**: Surgical stabilization allows for early mobilization and rehabilitation, which can improve functional outcomes, reduce complications related to prolonged bed rest (e.g., deep vein thrombosis, pressure sores), and enhance recovery.

4. Prevention of Complications Associated with Prolonged Immobilization

- **Reduced Risk of Bed Rest Complications**: Early surgery allows patients to avoid the complications associated with prolonged immobilization, such as pneumonia, muscle atrophy, and pressure sores.
- **Improved Rehabilitation Potential**: Patients who are surgically stabilized can typically engage in physical therapy and rehabilitation sooner, which supports muscle strength, endurance, and overall recovery.

5. Improved Long-Term Functional Outcomes

- **Enhanced Quality of Life**: Early surgical intervention has been shown to improve long-term outcomes by allowing patients to return to daily activities more quickly and with better functional capacity.
- **Lower Likelihood of Reoperation**: Early alignment and stabilization reduce the risk of progressive deformities and hardware failures that may necessitate additional surgery in the future.

6. Evidence from Studies

- Studies have shown that early surgical intervention (within 24–72 hours after injury) in patients with thoracic and lumbar fractures, particularly those with neurological deficits, is associated with better neurological recovery, decreased hospital

	stays, and improved overall outcomes compared to delayed surgery.<br>• The timing of surgery is most beneficial within the first 24–72 hours for neurological recovery, as this period allows for the best chance of mitigating secondary spinal cord injury from edema and inflammation.<br>**When Early Surgery May Not Be Indicated**<br>• **Medically Unstable Patients**: In patients who are medically unstable or have life-threatening injuries, stabilizing other critical conditions may take priority before spinal surgery.<br>• **Stable Fractures without Neurological Compromise**: For stable fractures without neurological involvement, non-surgical management may be effective, and early surgery may not significantly alter outcomes.<br>**Summary**<br>Early surgical intervention in patients with thoracic and lumbar fractures, particularly those with instability, neurological deficits, or risk of deformity, generally leads to better outcomes. By providing timely decompression, stabilization, and alignment correction, early surgery enhances neurological recovery, pain relief, functional mobility, and quality of life. Each case should be evaluated individually, considering the patient's overall health, fracture characteristics, and potential risks.
22. Does the choice of surgical approach (anterior, posterior, or combined anterior-posterior) improve clinical outcomes in patients with thoracic and lumbar fractures?<br>虽然跟前述的 9 给出了相似的建议，但是最初的结论是错误的。	Yes, the choice of surgical approach (anterior, posterior, or combined anterior-posterior) can significantly influence clinical outcomes in patients with thoracic and lumbar fractures. The optimal approach depends on the fracture characteristics, spinal stability, neurological involvement, and goals of the surgery. Here's how each approach impacts clinical outcomes:<br>**1. Posterior Approach**<br>• **Indications**: The posterior approach is widely used for thoracolumbar fractures and is generally preferred for unstable fractures, ligamentous injuries, and fractures requiring extensive stabilization. It is also ideal for fracture-dislocations and cases where posterior ligamentous complex (PLC) integrity is compromised.<br>• **Outcomes**:<br>○ **Effective Stabilization**: The posterior approach provides robust stabilization with pedicle screw and rod fixation, which can enhance spinal alignment and stability.<br>○ **Shorter Recovery Times**: Posterior approaches are typically associated with shorter surgical times and less invasive exposure compared to the anterior approach, which can reduce recovery time.<br>○ **Limitations**: While effective for stabilization, the posterior approach is less effective in cases with significant

anterior compression, such as burst fractures with retropulsed bone fragments, where direct decompression may be needed.

- **Overall**: The posterior approach generally offers excellent outcomes for stability, especially in cases requiring multi-level stabilization. However, it may be limited in achieving adequate decompression if anterior structures are involved.

2. Anterior Approach

- **Indications**: The anterior approach is commonly chosen for fractures with significant vertebral body involvement, severe burst fractures, or those with retropulsed bone fragments causing spinal cord compression.
- **Outcomes**:
  - **Direct Decompression**: The anterior approach allows for direct decompression by removing retropulsed bone fragments or damaged discs, which can improve neurological outcomes in patients with severe anterior compression.
  - **Enhanced Vertebral Body Support**: By placing a structural graft or cage, the anterior approach restores vertebral height and maintains alignment, which is particularly beneficial for restoring stability in cases of vertebral body collapse.
  - **Less Effective for Posterior Instability**: The anterior approach does not address posterior instability and is often inadequate when the posterior ligamentous complex is compromised.
- **Overall**: The anterior approach is ideal for improving outcomes in cases where anterior decompression and vertebral body support are needed, particularly for burst fractures with canal compromise. However, it may require supplementation with posterior fixation in more complex cases.

3. Combined Anterior-Posterior Approach

- **Indications**: The combined approach is usually reserved for complex, unstable fractures that require both anterior decompression and posterior stabilization, such as fracture-dislocations, severe burst fractures with both anterior and posterior instability, or injuries with significant kyphotic deformity.
- **Outcomes**:
  - **Comprehensive Stability**: The combined approach provides the highest level of stabilization, allowing correction of both anterior and posterior instability. This leads to a lower risk of future deformities or hardware failure.

	◦ **Improved Neurological Recovery**: The combined approach offers both direct decompression of neural elements and robust stabilization, which can improve neurological outcomes, especially in cases of multi-level or complex fractures.<br>◦ **Limitations**: The combined approach is more invasive, requires a longer surgical time, and is associated with a higher risk of complications, including blood loss and infection.<br>• **Overall**: Although more complex, the combined approach is highly effective in achieving long-term stability and alignment in cases with severe instability, extensive vertebral damage, or significant neurological compromise. It may improve functional and neurological outcomes but carries higher operative risks.<br>**Factors Influencing Choice of Approach and Outcomes**<br>• **Fracture Type and Stability**: Burst fractures with retropulsed fragments or vertebral body collapse often benefit from an anterior or combined approach, while fractures with posterior ligamentous injury are well-suited to a posterior or combined approach.<br>• **Neurological Compromise**: Patients with neurological deficits from anterior compression may achieve better outcomes with anterior or combined approaches for direct decompression, while those with primarily posterior compression may benefit from a posterior approach.<br>• **Surgeon Expertise and Patient Condition**: The choice of approach also depends on the surgeon's expertise, as well as patient-specific factors like bone quality, age, and medical stability, which may impact outcomes and recovery.<br>**Summary**<br>The choice of surgical approach can indeed improve clinical outcomes in patients with thoracic and lumbar fractures. Each approach has specific benefits depending on the type and complexity of the fracture:<br>• **Posterior Approach**: Preferred for stabilization, especially in posterior instability cases.<br>• **Anterior Approach**: Optimal for fractures with significant anterior compression requiring decompression and vertebral body support.<br>• **Combined Approach**: Provides the most comprehensive stability and decompression but is reserved for complex, unstable fractures due to its invasiveness.<br>Selecting the appropriate approach based on the patient's specific injury characteristics and overall health can optimize surgical

	outcomes, improve neurological recovery, maintain spinal alignment, and prevent complications.
23.Are there radiographic findings in patients with traumatic thoracolumbar fractures that can predict the need for surgical intervention?	Yes, certain radiographic findings in patients with traumatic thoracolumbar fractures can help predict the need for surgical intervention. These findings provide critical information about the stability of the fracture, the degree of spinal canal compromise, and the likelihood of neurological involvement. Here are the key radiographic indicators that may predict the need for surgery:<br>**1. Fracture Pattern and Morphology**<br>• **Burst Fractures**: Burst fractures, which involve the vertebral body shattering and retropulsion of bone fragments into the spinal canal, are strong predictors for surgery. They pose a high risk of spinal canal compromise and neurological injury, often necessitating decompression and stabilization.<br>• **Chance Fractures (Flexion-Distraction Injuries)**: Chance fractures, caused by flexion-distraction forces, involve posterior ligamentous complex (PLC) injury and usually require surgical stabilization due to their inherent instability.<br>• **Fracture-Dislocations**: These injuries involve displacement or dislocation of the vertebrae, which is highly unstable and typically requires surgical intervention to realign and stabilize the spine.<br>**2. Loss of Vertebral Body Height**<br>• **Severe Compression**: Loss of vertebral body height by more than 50% is often associated with instability and kyphotic deformity, which increases the likelihood of surgical intervention.<br>• **Kyphotic Deformity**: Significant anterior wedging or collapse that leads to kyphotic angulation (greater than 20 degrees) can indicate the need for surgical correction to restore alignment and prevent further deformity.<br>**3. Posterior Ligamentous Complex (PLC) Injury**<br>• **Evidence of PLC Disruption**: MRI findings, such as increased signal intensity in the posterior ligaments or torn ligaments, indicate disruption of the PLC. This injury suggests instability and strongly predicts the need for surgical stabilization.<br>• **Indirect Signs on X-ray/CT**: Widening of the interspinous distance, gapping at the facet joints, or posterior element fractures on X-ray or CT can suggest PLC involvement, which often requires surgical intervention.<br>**4. Spinal Canal Compromise**<br>• **Retropulsed Bone Fragments**: CT or MRI showing retropulsed bone fragments impinging on the spinal canal is a common indicator for surgery, especially if there is significant spinal cord or nerve root compression.<br>• **Canal Encroachment Greater than 50%**: When retropulsed fragments occupy more than 50% of the spinal canal, the risk of

neurological compromise increases, and decompressive surgery is often warranted.

5. Neurological Compromise

- **Compression of Neural Elements**: MRI findings indicating direct compression of the spinal cord or cauda equina by bone fragments, hematoma, or disc material often necessitate surgical decompression.
- **Presence of Neurological Deficits**: Even without MRI evidence, if neurological symptoms (e.g., weakness, numbness) correlate with a radiographic finding of compression or instability, surgical intervention is likely indicated to prevent further neurological deterioration.

6. Angular Deformity

- **Kyphosis or Segmental Angulation**: Significant angular deformity or kyphosis (typically greater than 20 degrees) at the fracture site often requires surgery to restore alignment, especially in the thoracolumbar junction, where such deformities can progress and cause chronic pain or functional impairment.

7. Thoracolumbar Injury Classification and Severity Score (TLICS)

- **High TLICS Score**: The TLICS system is commonly used to guide surgical decision-making based on radiographic findings and clinical assessment:
  - **Injury Morphology**: Compression (1 point), burst (2 points), translation/rotation (3 points), and distraction (4 points).
  - **PLC Integrity**: Intact (0 points), indeterminate (2 points), disrupted (3 points).
  - **Neurological Status**: Intact (0 points), nerve root injury (2 points), complete cord injury (2 points), incomplete cord injury (3 points).
- A TLICS score of **4 or higher** typically suggests that surgical intervention may be necessary, based on factors such as injury morphology, PLC disruption, and neurological involvement.

8. Other Indicators

- **Interpedicular Widening**: An increased interpedicular distance on X-ray or CT can suggest severe vertebral damage, instability, or potential PLC injury, all of which often require surgical stabilization.
- **Disc Space Involvement**: Compression or disruption of the adjacent disc space, especially in high-energy fractures, can indicate increased instability, necessitating surgery.

	**Summary**<br>Radiographic findings that predict the need for surgical intervention in thoracolumbar fractures include:<br>• **Burst or complex fractures** (e.g., Chance fractures, fracture-dislocations)<br>• **Significant vertebral body height loss** (more than 50%) or severe kyphotic angulation<br>• **Posterior ligamentous complex (PLC) injury**<br>• **Spinal canal compromise** (retropulsed fragments or significant canal encroachment)<br>• **Angular deformity** (kyphosis or segmental angulation greater than 20 degrees)<br>• **High TLICS score** (4 or higher)<br>These radiographic indicators help guide clinical decision-making by identifying cases with instability, risk of neurological compromise, or deformity progression, all of which often require surgical intervention to optimize outcomes and prevent complications.
24.Are there radiographic findings in patients with traumatic thoracolumbar fractures that can assist in predicting clinical outcomes?	Yes, certain radiographic findings in patients with traumatic thoracolumbar fractures can help predict clinical outcomes, including neurological recovery, stability, pain, and functional prognosis. These findings provide insights into the severity, stability, and potential complications of the fracture. Here are the key radiographic indicators that can assist in predicting clinical outcomes:<br>**1. Fracture Pattern and Morphology**<br>• **Burst Fractures**: Patients with burst fractures, especially those with retropulsed bone fragments, have a higher risk of spinal canal compromise and neurological deficits, which can adversely affect clinical outcomes. Burst fractures with significant canal compromise are often associated with a poorer prognosis in terms of neurological recovery.<br>• **Chance Fractures (Flexion-Distraction Injuries)**: Chance fractures (resulting from flexion-distraction forces) involving ligamentous injury tend to be more unstable and are associated with worse outcomes if not surgically stabilized, as they have a higher risk of chronic pain and deformity.<br>**2. Loss of Vertebral Body Height and Kyphotic Deformity**<br>• **Degree of Compression**: Greater vertebral body height loss, typically over 50%, is associated with poor clinical outcomes, including an increased risk of post-traumatic kyphosis, chronic back pain, and long-term disability.<br>• **Kyphotic Angle**: A significant kyphotic angle (generally >20 degrees) at the injury site is a predictor of poor outcomes, as kyphotic deformities are linked to persistent pain, functional limitations, and potential neurological compromise. Patients with substantial kyphotic deformity may require corrective surgery to improve outcomes.

	**3. Posterior Ligamentous Complex (PLC) Injury**<br>• **PLC Disruption**: MRI findings showing disruption of the posterior ligamentous complex (PLC), including the interspinous and supraspinous ligaments, indicate spinal instability. PLC injury is a strong predictor of poor outcomes, as instability can lead to progressive deformity, pain, and potentially further neurological deterioration.<br>• **Radiographic Signs of PLC Injury**: On X-ray or CT, indirect signs of PLC injury, such as widening of the interspinous distance, facet joint gapping, or posterior element fractures, also predict poor long-term outcomes if left untreated.<br>**4. Spinal Canal Compromise**<br>• **Canal Encroachment by Retropulsed Fragments**: CT or MRI findings of retropulsed bone fragments that encroach upon the spinal canal are associated with a higher likelihood of neurological deficits and poorer neurological recovery. Greater than 50% spinal canal compromise is particularly concerning and is linked to an increased risk of long-term neurological impairment.<br>• **Degree of Compression on Neural Elements**: MRI evidence of direct compression on the spinal cord or cauda equina by bone or disc material correlates with worse neurological and functional outcomes.<br>**5. Neurological Status Correlation with Radiographic Findings**<br>• **Correlation with MRI Findings**: Radiographic findings, such as the presence of spinal cord compression or myelomalacia (softening of the spinal cord tissue) on MRI, often correlate with severe neurological impairment and predict poor neurological recovery in patients with incomplete injuries.<br>• **Bone Edema on MRI**: Extensive bone marrow edema on MRI may indicate high-impact trauma and severe structural injury, potentially leading to prolonged pain and delayed recovery.<br>**6. Angular Deformity and Alignment**<br>• **Kyphotic and Rotational Deformities**: Angular or rotational deformities are associated with a higher likelihood of long-term pain, difficulty in spinal alignment, and post-traumatic kyphosis, all of which can impact quality of life and functional outcomes.<br>• **Sagittal Alignment**: Poor sagittal alignment, particularly in the thoracolumbar junction, is often associated with an increased risk of progressive deformity, chronic pain, and functional limitations if not corrected.<br>**7. Thoracolumbar Injury Classification and Severity Score (TLICS)**

	- **Higher TLICS Scores**: A higher TLICS score (usually 4 or above) is associated with worse clinical outcomes due to the increased likelihood of instability and neurological involvement. TLICS takes into account fracture morphology, PLC integrity, and neurological status, making it a useful predictor of prognosis.<br>- **Need for Surgery**: Higher TLICS scores often lead to surgical intervention, which may improve stability and outcomes but indicate a more severe initial injury.<br>**8. Disc Space Involvement**<br>- **Compression of Adjacent Disc Space**: Compression or narrowing of the disc space adjacent to the fracture suggests a more severe impact and structural instability, which can be associated with poor outcomes, including chronic pain and disc degeneration.<br>- **Disrupted Discs**: Disc disruption can also increase the likelihood of post-traumatic arthritis and degenerative changes, which contribute to long-term pain and functional impairment.<br>**9. Interpedicular Distance Widening**<br>- **Increased Interpedicular Distance**: On X-ray or CT, a widened interpedicular distance at the fracture level suggests significant structural damage and potential ligamentous disruption, both of which are predictors of poor outcomes if not stabilized.<br>**Summary**<br>Radiographic findings that can help predict clinical outcomes in patients with traumatic thoracolumbar fractures include:<br>- **Fracture morphology** (burst, Chance, or fracture-dislocations)<br>- **Vertebral body height loss** and **kyphotic deformity**<br>- **Posterior ligamentous complex (PLC) injury**<br>- **Spinal canal compromise** and degree of neural compression<br>- **Angular deformity and poor sagittal alignment**<br>- **High TLICS score**<br>- **Disc space involvement** and **interpedicular widening**<br>These indicators reflect the severity of the injury, stability, and the likelihood of complications such as pain, deformity, and neurological deficits. Recognizing these radiographic predictors allows clinicians to identify patients at higher risk of poor outcomes, guiding both

	the need for surgical intervention and a more aggressive rehabilitation approach.
25.Does routine screening for DVT prevent PE (or VTE-associated morbidity and mortality) in patients with thoracic and lumbar fractures?	Routine screening for deep vein thrombosis (DVT) in patients with thoracic and lumbar fractures has been a topic of debate regarding its effectiveness in preventing pulmonary embolism (PE) and reducing VTE-associated morbidity and mortality. Here's a detailed analysis of the role of routine screening in this context:<br>**1. Understanding the Relationship Between DVT and PE**<br>• **DVT and PE Connection**: DVT occurs when a blood clot forms in a deep vein, typically in the legs. If a clot dislodges, it can travel to the lungs and cause a pulmonary embolism (PE), which is a serious and potentially life-threatening condition.<br>• **Risk Factors**: Patients with thoracic and lumbar fractures are at increased risk for DVT due to immobility, trauma, and potential surgical interventions, which can all contribute to venous stasis and hypercoagulability.<br>**2. Efficacy of Routine Screening for DVT**<br>• **Screening vs. Prophylaxis**: Routine screening (e.g., ultrasound) for DVT aims to detect clots early, but it does not directly prevent the occurrence of DVT or PE. Prophylaxis, on the other hand, involves preventive measures (like anticoagulation or mechanical devices) to reduce the risk of clot formation.<br>• **Limited Impact on PE Prevention**: Evidence suggests that while routine screening can detect DVTs that may otherwise go unnoticed, it does not significantly decrease the incidence of PE. Many PEs can occur without detectable prior DVT or can originate from other venous sites (e.g., pelvic veins) not routinely screened.<br>**3. Benefits of DVT Screening**<br>• **Early Detection**: Routine screening may be beneficial in identifying asymptomatic DVTs, allowing for timely intervention before the clots result in PE. However, the effectiveness of this approach depends on the subsequent management of detected DVTs.<br>• **Targeted Interventions**: If screening reveals DVTs, initiating appropriate anticoagulation can help prevent the progression to PE. Thus, the value of routine screening may lie more in its ability to guide treatment rather than in preventing DVT occurrence.<br>**4. Risks and Limitations of Routine Screening**<br>• **False Positives**: Routine screening can yield false-positive results, leading to unnecessary anxiety and potential overtreatment (such as anticoagulation), which carries its own risks, including bleeding complications.

	• **Resource Intensity**: Routine ultrasound screening requires significant healthcare resources, and the cost-effectiveness of this approach in the absence of widespread clinical benefit is debated.<br>**5. Current Recommendations and Guidelines**<br>• **Focus on Prophylaxis**: Most clinical guidelines emphasize the importance of prophylaxis (e.g., anticoagulants or mechanical compression devices) over routine screening. The emphasis is on preventing DVT formation rather than solely relying on screening to detect it.<br>• **Selective Screening**: Screening may be indicated in patients with specific risk factors or those who develop signs and symptoms suggestive of DVT, rather than as a blanket approach for all patients with thoracic and lumbar fractures.<br>**6. Conclusions from Clinical Evidence**<br>• **Lack of Strong Evidence for Routine Screening**: There is insufficient evidence to support routine DVT screening as a method to prevent PE in thoracic and lumbar fracture patients. Prophylactic measures remain the primary strategy for reducing VTE risk.<br>• **Recommendation for Targeted Approach**: A targeted approach to screening, focused on high-risk patients or those with clinical signs of DVT, may be more beneficial than routine screening for all patients, coupled with effective prophylactic measures.<br>**Summary of Key Points**<br>• **Routine screening for DVT** does not significantly prevent PE or reduce VTE-associated morbidity and mortality in patients with thoracic and lumbar fractures.<br>• **Prophylaxis** is critical for reducing the risk of DVT and PE.<br>• **Selective screening** may be appropriate for certain high-risk patients, but broad routine screening is not generally recommended.<br>**Conclusion**<br>In summary, while routine screening for DVT can help in detecting asymptomatic clots, it does not effectively prevent pulmonary embolism or reduce VTE-related complications in patients with thoracic and lumbar fractures. The focus should remain on implementing effective prophylactic measures to reduce the risk of DVT and PE, while selective screening may be beneficial in high-risk individuals.

26.For patients with thoracic and lumbar fractures, is one regimen of VTE prophylaxis superior to others with respect to prevention of PE (or VTE-associated morbidity and mortality)?	In patients with thoracic and lumbar fractures, certain regimens of venous thromboembolism (VTE) prophylaxis are generally preferred based on their efficacy in reducing VTE-related morbidity and mortality, including the prevention of pulmonary embolism (PE). However, the superiority of one regimen over another can vary depending on the individual patient's risk factors, potential for bleeding, and the feasibility of each regimen. Here's a breakdown of the main prophylaxis regimens and considerations:<br>**1. Low Molecular Weight Heparin (LMWH)**<br>● **Effectiveness**: LMWH is widely considered the gold standard for VTE prophylaxis in trauma patients, including those with thoracic and lumbar fractures. It has been shown to be effective in reducing the incidence of DVT and PE.<br>● **Safety Profile**: LMWH has a relatively predictable pharmacokinetic profile, which reduces the need for frequent monitoring and allows for consistent dosing, making it convenient and safe for long-term use.<br>● **Advantages**:<br>○ **Reduced Risk of Major Bleeding**: LMWH is associated with a lower risk of major bleeding compared to unfractionated heparin (UFH).<br>○ **Once or Twice Daily Dosing**: LMWH is typically administered once or twice daily, which is convenient for both inpatients and outpatients.<br>● **Limitations**: LMWH requires adjustment in patients with renal impairment, and its subcutaneous administration may be challenging for patients with needle aversion or those in home care settings.<br>**2. Unfractionated Heparin (UFH)**<br>● **Effectiveness**: UFH is effective in reducing VTE incidence but may be less effective than LMWH in preventing PE in high-risk trauma patients.<br>● **Safety Profile**: UFH requires frequent monitoring of activated partial thromboplastin time (aPTT) due to its variable pharmacokinetics and is associated with a higher risk of heparin-induced thrombocytopenia (HIT) compared to LMWH.<br>● **Advantages**:<br>○ **Short Half-Life**: UFH's short half-life makes it easy to discontinue quickly if surgical intervention is required, making it a useful option in perioperative settings.<br>○ **Reversibility**: UFH can be fully reversed with protamine sulfate, which can be beneficial if there is a concern about bleeding risk.

	• **Limitations**: Due to the need for frequent monitoring and increased bleeding risk, UFH is less commonly used as a primary prophylactic agent for patients with thoracic and lumbar fractures.<br>**3. Direct Oral Anticoagulants (DOACs)**<br>• **Effectiveness**: DOACs, such as rivaroxaban and apixaban, are increasingly being used for VTE prophylaxis in general orthopedic and trauma patients. However, there is limited evidence on their safety and efficacy specifically in spinal trauma patients, as they are typically avoided in high-risk bleeding situations.<br>• **Safety Profile**: DOACs have a lower bleeding risk than warfarin and do not require routine monitoring. However, they are difficult to reverse in cases of acute bleeding.<br>• **Advantages**:<br>○ **Convenient Dosing**: DOACs offer convenient oral administration, which may be appealing for long-term prophylaxis after discharge.<br>○ **No Routine Monitoring**: DOACs eliminate the need for frequent blood monitoring, making them easier to manage in outpatient settings.<br>• **Limitations**: Limited data in spinal trauma and potential bleeding concerns mean DOACs are typically not the first-line option in patients with thoracic or lumbar fractures.<br>**4. Aspirin**<br>• **Effectiveness**: Aspirin is less effective than anticoagulants (LMWH, UFH, and DOACs) in preventing DVT and PE and is generally not recommended as a standalone VTE prophylactic agent in high-risk patients with thoracic and lumbar fractures.<br>• **Advantages**: Due to its safety profile, low cost, and ease of use, aspirin may be considered as an adjunct in patients with a high bleeding risk where anticoagulants are contraindicated.<br>• **Limitations**: The efficacy of aspirin in preventing VTE is inferior to other anticoagulants, and it should not be used as the sole prophylactic method in high-risk trauma patients.<br>**5. Mechanical Prophylaxis (Intermittent Pneumatic Compression and Graduated Compression Stockings)**<br>• **Effectiveness**: Mechanical prophylaxis, including intermittent pneumatic compression (IPC) devices and graduated compression stockings (GCS), can reduce venous stasis but is not as effective as pharmacologic prophylaxis in preventing DVT and PE.

- **Advantages**:
    - **Low Bleeding Risk**: Mechanical methods pose no risk of bleeding, making them ideal adjuncts for patients with a high bleeding risk or as a supplement to pharmacologic prophylaxis.
    - **Use in Combination with Anticoagulation**: Combining mechanical prophylaxis with anticoagulants can be more effective in high-risk patients.
- **Limitations**: Mechanical prophylaxis alone is insufficient for preventing VTE in high-risk trauma patients, and compliance can be challenging in immobile or noncompliant patients.

6. Combined Pharmacologic and Mechanical Prophylaxis

- **Superior Efficacy in High-Risk Patients**: For high-risk patients, including those with multiple fractures or prolonged immobility, combining pharmacologic prophylaxis (typically LMWH) with mechanical methods (e.g., IPC) has been shown to reduce the incidence of DVT and PE more effectively than either method alone.
- **Improved Outcomes**: Studies indicate that the combination of LMWH and mechanical prophylaxis is associated with lower rates of VTE and may reduce VTE-related morbidity and mortality in trauma patients.

Summary of Preferred Regimens for Thoracic and Lumbar Fractures

1. **Low Molecular Weight Heparin (LMWH)**: Currently considered the preferred pharmacologic option due to its balance of efficacy, safety, and convenience in high-risk trauma patients, including those with thoracic and lumbar fractures.
2. **Unfractionated Heparin (UFH)**: A reasonable alternative in cases where frequent monitoring is feasible or when quick reversibility is necessary.
3. **Combined Pharmacologic and Mechanical Prophylaxis**: For high-risk patients, using LMWH in conjunction with mechanical methods like IPC is generally superior in reducing VTE events and is recommended when bleeding risk is manageable.
4. **Aspirin and DOACs**: Aspirin is generally not recommended as a primary method for VTE prophylaxis in high-risk patients due to limited efficacy. DOACs are used cautiously due to limited evidence in spinal trauma and are typically not first-line in this setting.

Conclusion

While LMWH is generally the preferred option for VTE prophylaxis in patients with thoracic and lumbar fractures, combining LMWH

	with mechanical methods offers enhanced protection in high-risk patients. UFH may be used in certain situations, such as when rapid reversibility is needed. DOACs and aspirin are less favored due to limited data in spinal trauma and lower efficacy in preventing VTE. Ultimately, the best regimen should be individualized, balancing the patient's bleeding risk, VTE risk, and practical considerations for long-term management.
27.Is there a specific treatment regimen for documented VTE that provides fewer complications than other treatments in patients with thoracic and lumbar fractures?	In patients with thoracic and lumbar fractures who develop a documented venous thromboembolism (VTE), the choice of treatment regimen must balance effective VTE management with the potential for bleeding and other complications, especially in the context of spinal trauma. Several treatment regimens are commonly used, each with different risk profiles. Here's an analysis of the most widely used regimens and their complication rates:<br>**1. Low Molecular Weight Heparin (LMWH)**<br>● **Effectiveness**: LMWH is often preferred for treating VTE in trauma patients due to its predictable pharmacokinetics, which provide effective anticoagulation with a lower risk of bleeding than some alternatives.<br>● **Safety Profile**:<br>○ **Lower Risk of Major Bleeding**: LMWH has a lower risk of major bleeding compared to unfractionated heparin (UFH) and can be used safely in trauma patients, including those with spinal fractures, unless contraindications exist.<br>○ **Reduced Risk of Heparin-Induced Thrombocytopenia (HIT)**: LMWH has a lower incidence of HIT than UFH, making it safer for long-term treatment in patients requiring extended anticoagulation.<br>● **Complications**: The main complications with LMWH include minor bleeding and potential renal accumulation in patients with kidney impairment, necessitating dose adjustment in such cases.<br>● **Advantages**: LMWH can be administered subcutaneously once or twice daily, which is convenient and allows patients to be treated in outpatient settings when appropriate.<br>**2. Unfractionated Heparin (UFH)**<br>● **Effectiveness**: UFH is effective for VTE treatment and is typically administered as an intravenous infusion, allowing for rapid anticoagulation and close monitoring, which is beneficial for patients with fluctuating bleeding risk.<br>● **Safety Profile**:

	○ **Higher Risk of HIT**: UFH has a higher incidence of HIT than LMWH, which can complicate treatment if HIT develops.<br>○ **Increased Bleeding Risk**: Compared to LMWH, UFH has a slightly higher bleeding risk, especially with continuous infusion, but its short half-life makes it easy to discontinue rapidly if needed.<br>● **Complications**: The need for frequent monitoring (aPTT) and higher bleeding risk make UFH less desirable for long-term management, but it remains valuable for initial treatment, especially in high-risk cases where rapid reversibility is needed.<br>● **Advantages**: UFH's short half-life and full reversibility with protamine sulfate make it useful in patients who may require urgent surgical intervention.<br>**3. Direct Oral Anticoagulants (DOACs)**<br>● **Effectiveness**: DOACs, including rivaroxaban and apixaban, are increasingly used for VTE treatment due to their convenience, fixed dosing, and similar efficacy to traditional anticoagulants.<br>● **Safety Profile**:<br>○ **Lower Bleeding Risk in General**: DOACs tend to have a lower bleeding risk than warfarin, but data specifically in spinal trauma patients are limited, so caution is warranted.<br>○ **Challenges with Reversibility**: Although newer reversal agents are available for some DOACs, the reversibility is not as immediate as with UFH, which can be a concern in patients at risk of needing urgent intervention.<br>● **Complications**: DOACs can be challenging in patients with renal impairment, as they are partially renally cleared, necessitating dose adjustment or avoidance in severe kidney disease.<br>● **Advantages**: DOACs offer convenient oral administration and do not require regular monitoring, making them suitable for long-term management if the patient's bleeding risk is stable.<br>**4. Warfarin (Vitamin K Antagonist)**<br>● **Effectiveness**: Warfarin is effective for long-term VTE treatment, but it has been largely replaced by LMWH and DOACs due to the need for frequent monitoring and a slower onset of action.<br>● **Safety Profile**:<br>○ **Higher Bleeding Risk**: Warfarin carries a higher risk of major bleeding, particularly in trauma patients, and requires close monitoring with INR to maintain therapeutic levels.

	○ **Drug and Dietary Interactions**: Warfarin's effects are influenced by dietary vitamin K and other medications, which can complicate management and increase the risk of both bleeding and clotting.<br>• **Complications**: Frequent INR monitoring and dose adjustments are required, which may be challenging in a trauma patient. It also takes several days to reach therapeutic levels, so bridging with LMWH or UFH is typically necessary.<br>• **Advantages**: Warfarin's effects can be reversed with vitamin K, which is beneficial if bleeding occurs, but its slower onset and interaction profile make it less favored in trauma patients.<br>**5. Inferior Vena Cava (IVC) Filter Placement**<br>• **Effectiveness**: IVC filters are not a primary treatment for VTE but may be used as an adjunct in patients at high risk for PE who cannot receive anticoagulation due to bleeding risks.<br>• **Safety Profile**:<br>○ **Risk of Filter-Related Complications**: Complications include filter migration, thrombosis, and, if left in place long-term, potential for VTE recurrence above the filter.<br>○ **Limited Impact on Mortality**: IVC filters reduce the risk of PE but do not reduce overall mortality related to VTE.<br>• **Complications**: Filters are typically temporary and should be removed when anticoagulation becomes feasible to avoid long-term risks.<br>• **Advantages**: They provide a non-anticoagulant method for PE prevention in patients with contraindications to anticoagulation.<br>**Summary of Preferred Regimen Based on Complication Profile**<br>1. **Low Molecular Weight Heparin (LMWH)**: Generally considered the preferred treatment for documented VTE in patients with thoracic and lumbar fractures due to its efficacy, safety profile, lower bleeding risk, and lower incidence of HIT. It is effective for both inpatient and outpatient management and has fewer complications overall.<br>2. **Unfractionated Heparin (UFH)**: Useful for initial management in high-risk patients who may require close monitoring or rapid reversibility. Its higher risk of HIT and need for frequent monitoring make it less ideal for long-term use.<br>3. **Direct Oral Anticoagulants (DOACs)**: Effective and convenient for long-term VTE management in patients who are stable and at low risk of needing surgical intervention, but data on safety in spinal trauma are limited, and reversibility may be a concern.

	4. **Warfarin**: An alternative for long-term VTE prevention but generally less favored due to the need for monitoring and a higher bleeding risk profile. It may be used if DOACs are not suitable.<br>5. **IVC Filters**: Considered only for patients with contraindications to anticoagulation who are at high risk of PE. Filters should be removed once anticoagulation can be safely started.<br>**Conclusion**<br>For patients with documented VTE and thoracic or lumbar fractures, **LMWH is generally the regimen with the fewest complications** and is preferred for both efficacy and safety. UFH may be used initially in high-risk cases where close monitoring is needed, while DOACs may be an option for stable patients in the long term. IVC filters are reserved for cases where anticoagulation is contraindicated. The choice should be individualized, considering the patient's risk profile, renal function, and potential need for future surgical intervention.
28.Does the administration of a specific pharmacologic agent (e.g., methylprednisolone) improve clinical outcomes in patients with thoracic and lumbar fractures and spinal cord injury?	The administration of specific pharmacologic agents, such as methylprednisolone, in patients with thoracic and lumbar fractures and spinal cord injury (SCI) has been a topic of significant debate. While methylprednisolone was once widely used in the acute management of spinal cord injuries, current evidence on its effectiveness and safety is mixed, and its use is now controversial due to potential risks.<br>**1. Methylprednisolone (Corticosteroid Therapy)**<br>• **Historical Use**: Methylprednisolone was initially recommended following spinal cord injury based on the National Acute Spinal Cord Injury Study (NASCIS) trials in the 1990s. These studies suggested that high-dose methylprednisolone given within 8 hours of injury might improve neurological outcomes.<br>• **Current Evidence and Guidelines**:<br>○ **Limited Efficacy**: Subsequent research and analyses have questioned the efficacy of methylprednisolone in improving long-term neurological outcomes, especially for thoracic and lumbar injuries, where the potential for functional recovery may differ compared to cervical SCI.<br>○ **High Risk of Complications**: High-dose corticosteroids like methylprednisolone are associated with significant adverse effects, including increased risk of infections, gastrointestinal bleeding, delayed wound healing, sepsis, and even mortality in some cases. The potential risks are often deemed to outweigh the benefits.<br>• **Guideline Recommendations**: Major organizations, such as the American Association of Neurological Surgeons (AANS) and Congress of Neurological Surgeons (CNS), now advise against routine use of high-dose methylprednisolone for acute spinal

	cord injuries due to the lack of consistent benefit and the high risk of complications.<br>**2. Other Pharmacologic Agents Being Studied**<br>• **GM-1 Ganglioside (Sygen)**: Previously studied for its neuroprotective properties in SCI, Sygen showed promise in early trials but ultimately did not demonstrate consistent benefit in larger studies. It is no longer widely used for SCI management.<br>• **Minocycline**: This antibiotic with anti-inflammatory and neuroprotective properties has been studied for its potential to reduce secondary injury in SCI. Preliminary studies show some promise, but larger trials are needed to confirm its efficacy and safety in clinical practice.<br>• **Riluzole**: An agent used in amyotrophic lateral sclerosis (ALS), riluzole has been studied for its potential neuroprotective effects in SCI by inhibiting glutamate release. Early studies suggest some benefit, but further research is needed, and it is not yet a standard treatment.<br>• **N-Acetylcysteine (NAC)**: NAC has antioxidant properties that may help reduce oxidative stress in SCI. While promising in animal studies, it lacks sufficient evidence from human trials to be recommended for routine clinical use.<br>**3. Current Standard of Care and Alternative Approaches**<br>• **Focus on Surgical and Supportive Interventions**: The primary management for thoracic and lumbar fractures with SCI typically involves timely surgical decompression and stabilization, followed by supportive care, physical therapy, and rehabilitation.<br>• **Neuroprotective Strategies Under Investigation**: Research continues on neuroprotective agents, but none have proven effective enough in clinical trials to become a part of standard practice. Ongoing studies focus on minimizing secondary injury through non-pharmacologic means, such as maintaining optimal blood pressure, oxygenation, and spinal alignment.<br>**4. Summary of Methylprednisolone Use and Current Best Practices**<br>• **Limited Role in SCI Management**: While methylprednisolone was once considered beneficial in SCI treatment, current evidence does not support its routine use due to the lack of consistent neurological improvement and high risk of severe complications.<br>• **Guidelines Recommend Against Routine Use**: Most modern guidelines, including those from the AANS and CNS, do not recommend high-dose methylprednisolone for acute thoracic and lumbar SCI due to safety concerns.<br>• **Focus on Early Surgical Intervention and Supportive Care**: The emphasis in current SCI management is on rapid surgical

	intervention for decompression and stabilization, followed by comprehensive supportive care and rehabilitation to maximize functional recovery.<br>**Conclusion**<br>There is currently no pharmacologic agent, including methylprednisolone, that has consistently demonstrated improved clinical outcomes in patients with thoracic and lumbar fractures with SCI. The risks of high-dose corticosteroids outweigh potential benefits, and they are no longer routinely recommended. Instead, early surgical intervention and supportive care remain the cornerstones of effective management for improving outcomes in these patients.
29.Does the surgical treatment of burst fractures of the thoracic and lumbar spine improve clinical outcomes compared to nonoperative treatment?	Yes, surgical treatment of burst fractures in the thoracic and lumbar spine can improve clinical outcomes compared to nonoperative treatment in certain cases, particularly when there is spinal instability, significant deformity, or neurological compromise. Here's a detailed look at when surgical treatment is beneficial and how it compares to nonoperative approaches:<br>**1. Indications for Surgical vs. Nonoperative Treatment**<br>● **Surgical Treatment**:<br>○ **Neurological Deficits**: Surgery is generally indicated for burst fractures with neurological deficits due to spinal canal compression, as it allows for decompression of neural elements.<br>○ **Significant Spinal Instability**: If the fracture results in spinal instability, as indicated by posterior ligamentous complex (PLC) disruption or excessive vertebral body collapse, surgery can restore stability, realign the spine, and prevent further deformity.<br>○ **Severe Kyphotic Deformity**: Patients with significant kyphotic angulation (usually >20–30 degrees) are often better treated with surgery to correct and maintain spinal alignment. Severe kyphosis can lead to chronic pain and functional limitations if left untreated.<br>○ **Intractable Pain**: Persistent, severe pain despite nonoperative management may be an indication for surgery to stabilize the fracture and alleviate discomfort.<br>● **Nonoperative Treatment**:<br>○ **Stable Burst Fractures**: In cases where the burst fracture is stable, meaning there is minimal vertebral body height loss, no significant kyphotic deformity, and intact PLC, nonoperative treatment can be effective.<br>○ **No Neurological Compromise**: For fractures without spinal cord or nerve root compression, nonoperative

	approaches are often sufficient, particularly when stability is not a concern.<br>○ **Patient Preference or Medical Contraindications**: Some patients may prefer nonoperative management or may be unsuitable candidates for surgery due to medical comorbidities.<br>**2. Outcomes with Surgical Treatment**<br>• **Improved Neurological Outcomes**: Surgery allows for direct decompression of the spinal canal by removing retropulsed bone fragments, which can improve neurological recovery in patients with deficits.<br>• **Enhanced Stability and Alignment**: Surgical fixation and fusion restore stability to the spine, maintain alignment, and prevent progressive kyphosis. This is especially important in unstable fractures where progressive deformity is a risk.<br>• **Pain Relief and Early Mobilization**: By stabilizing the fracture, surgery can reduce pain and allow for earlier mobilization, which improves recovery and reduces complications associated with prolonged bed rest, such as deep vein thrombosis (DVT) and pulmonary complications.<br>• **Lower Long-Term Complication Rates**: Surgical stabilization can reduce the risk of chronic pain and deformity, which are common complications of untreated or inadequately treated unstable fractures.<br>**3. Outcomes with Nonoperative Treatment**<br>• **Effective for Stable Fractures**: Nonoperative treatment, typically involving bracing and restricted activity, can be effective for stable fractures without neurological compromise or significant deformity. Stable burst fractures tend to heal well with conservative measures and do not require surgical intervention.<br>• **Avoidance of Surgical Risks**: Nonoperative management avoids the risks associated with surgery, such as infection, blood loss, and anesthesia complications. This is particularly advantageous for patients with comorbidities that increase surgical risk.<br>• **Risk of Progressive Deformity and Chronic Pain**: For some patients, nonoperative treatment may lead to progressive kyphotic deformity or chronic pain, especially if the fracture is less stable than initially thought. This is a concern in cases with borderline instability or unrecognized PLC injury.<br>**4. Comparative Studies and Evidence**<br>• **Neurological Recovery**: Studies generally support surgery in patients with neurological deficits due to burst fractures, as surgical decompression improves neurological outcomes more effectively than nonoperative care.

- **Spinal Alignment and Stability**: Surgical treatment has been shown to maintain spinal alignment better than nonoperative treatment, particularly in unstable fractures. Long-term studies suggest that patients with surgical fixation tend to have better spinal alignment and reduced kyphosis.
- **Pain and Functional Outcomes**: Surgical stabilization often leads to better pain control and functional outcomes in the long term for patients with unstable fractures or significant deformity.
- **Quality of Life**: In cases where surgery is indicated, studies indicate better quality of life scores and reduced disability compared to nonoperative treatment, as surgery can provide more reliable stability, alignment, and pain relief.

5. Limitations and Risks of Surgical Treatment

- **Surgical Risks**: Surgery carries risks of infection, blood loss, hardware failure, and adjacent segment degeneration over time, which must be weighed against the potential benefits.
- **Longer Recovery for Complex Procedures**: Complex procedures, especially those requiring combined anterior and posterior approaches, may have longer recovery times and increased risk of complications compared to conservative care.

Summary

In summary, surgical treatment is generally superior to nonoperative management for thoracic and lumbar burst fractures in patients with:

- Neurological deficits
- Spinal instability
- Significant kyphotic deformity
- Intractable pain despite conservative care

For stable burst fractures without neurological compromise, nonoperative treatment is often effective, avoiding surgical risks while still providing good outcomes.

Conclusion

Surgical treatment improves clinical outcomes in patients with thoracic and lumbar burst fractures when there is instability, neurological compromise, or risk of progressive deformity. Nonoperative treatment is a viable option for stable fractures without these risk factors, providing effective healing with lower risk. The decision should be individualized based on fracture characteristics, patient health, and the goals of treatment.

30.Does the surgical treatment of nonburst fractures of the thoracic and lumbar spine improve clinical outcomes compared to nonoperative treatment?	The surgical treatment of nonburst fractures of the thoracic and lumbar spine may improve clinical outcomes compared to nonoperative treatment in specific cases, but generally, the need for surgery is less frequent for these types of fractures. Nonburst fractures include stable compression fractures and minor fractures without significant vertebral body or ligamentous complex disruption. Here's an overview of how surgical and nonoperative treatments compare, and when surgery may be beneficial:<br>**1. Types of Nonburst Fractures and Their Typical Management**<br>● **Stable Compression Fractures**: These fractures involve minor anterior vertebral body compression without affecting the posterior vertebral body or posterior ligamentous complex (PLC). Stable compression fractures are usually managed nonoperatively with bracing, pain control, and activity modification.<br>● **Transverse Process Fractures**: These are generally stable fractures that rarely require surgery and are usually treated with conservative measures.<br>● **Minor Vertebral Fractures**: Small fractures without significant deformity or risk of neurological injury are typically stable and can heal effectively with nonoperative care.<br>**2. When Nonoperative Treatment is Preferred**<br>● **Stable Fractures Without Neurological Compromise**: Nonoperative treatment is typically the first choice for stable nonburst fractures, as these fractures generally have a low risk of progression and a good prognosis for healing without surgery.<br>● **Benefits of Nonoperative Treatment**:<br>○ **Avoids Surgical Risks**: Nonoperative management avoids the risks associated with surgery, such as infection, blood loss, and potential hardware complications.<br>○ **Sufficient for Stable Injuries**: Conservative treatment, including bracing and pain management, is often sufficient for stable fractures that don't compromise spinal stability or alignment.<br>● **Patient Suitability**: Nonoperative management is especially suitable for patients with comorbidities that increase surgical risk, as well as elderly patients who may tolerate surgery poorly.<br>**3. When Surgical Treatment May Be Beneficial**<br>● **Intractable Pain**: In some cases, despite conservative measures, patients may experience persistent, severe pain that affects their quality of life and ability to function. Surgery may be considered to stabilize the fracture and alleviate pain in these

	cases.<br>• **Progressive Kyphotic Deformity**: In rare cases, stable fractures can progress to significant kyphotic deformity, especially if there is underlying osteoporosis or poor bone quality. Surgery may be indicated to correct alignment and prevent further deformity.<br>• **Unstable Nonburst Fractures**: Certain nonburst fractures, such as fractures with subtle instability or occult ligamentous injury, may benefit from surgery. Examples include fractures with significant vertebral height loss (greater than 50%) or vertebral wedging with kyphotic angulation.<br>**4. Comparative Outcomes of Surgical vs. Nonoperative Treatment**<br>• **Pain Relief and Function**: In stable nonburst fractures, studies generally show that nonoperative treatment provides comparable pain relief and functional outcomes to surgical treatment, especially over the long term. Most patients achieve adequate recovery with conservative management.<br>• **Spinal Stability and Alignment**: For fractures that do not impact the posterior ligamentous complex, nonoperative treatment can maintain spinal stability and alignment. However, in cases with progressive deformity, surgery may help in restoring alignment and preventing chronic postural issues.<br>• **Quality of Life and Mobility**: Nonoperative treatment often allows patients to maintain good mobility and quality of life. Surgical intervention can improve outcomes in cases where pain and deformity limit function, but it is generally not necessary for stable nonburst fractures.<br>• **Complications and Recovery**: Surgical treatment carries higher risks of complications and a longer recovery period compared to conservative treatment, which is a significant factor in favor of nonoperative management for stable nonburst fractures.<br>**5. Guideline Recommendations**<br>• **Conservative Management as First-Line Treatment**: For stable nonburst fractures without neurological involvement or significant instability, conservative management (bracing, pain control, physical therapy) is the preferred approach.<br>• **Selective Surgical Indication**: Surgery may be indicated selectively in cases where conservative treatment fails to provide adequate pain relief, or in cases with progressive deformity, even though these instances are relatively rare.<br>**6. Specific Surgical Techniques Used When Surgery is Indicated**

	• **Vertebroplasty and Kyphoplasty**: For compression fractures with significant pain due to vertebral collapse (especially in osteoporotic patients), minimally invasive procedures like vertebroplasty or kyphoplasty can provide pain relief and restore some vertebral height.<br>• **Posterior Instrumentation**: In cases with subtle instability or progressive deformity, posterior instrumentation with pedicle screws may be used to restore stability and correct alignment.<br>• **Fusion**: In rare cases where there is a risk of progressive deformity, fusion may be considered to provide long-term stability.<br>**Summary**<br>For most patients with nonburst fractures of the thoracic and lumbar spine, **nonoperative treatment is typically sufficient and preferred**, offering good pain relief, functional recovery, and minimal complications. Surgical treatment does not generally improve outcomes for stable nonburst fractures and carries additional risks.<br>Surgery may be considered in specific cases of nonburst fractures when there is:<br>• **Intractable pain** not responsive to conservative management<br>• **Progressive deformity** or instability (rare in nonburst fractures)<br>• **Underlying osteoporosis** or other risk factors that could lead to further collapse<br>In summary, **nonoperative management is the standard approach** for stable nonburst fractures without instability or neurological compromise, with surgery reserved for selective cases where conservative treatment fails. This approach balances effective recovery with minimal risk, favoring functional outcomes and quality of life in most patients.
31.Does the addition of arthrodesis to instrumented fixation improve outcomes in patients with thoracic and lumbar burst fractures?	The addition of arthrodesis (spinal fusion) to instrumented fixation in patients with thoracic and lumbar burst fractures can improve outcomes in certain cases, but its benefits depend on specific fracture characteristics, stability, and the patient's overall condition. Arthrodesis is the surgical fusion of two or more vertebrae, intended to provide long-term stability and prevent movement at the fractured segment. Here's an analysis of when adding arthrodesis may be beneficial and how it compares to instrumented fixation alone.<br>**1. Indications for Adding Arthrodesis**<br>• **Unstable Burst Fractures**: For burst fractures with significant instability, particularly those involving posterior ligamentous complex (PLC) injury or vertebral body collapse, arthrodesis can provide additional stability and prevent future complications, such as progressive deformity.

	- **Severe Kyphotic Deformity**: In patients with significant kyphotic angulation or those at risk of developing kyphosis, fusion helps maintain alignment and prevents further deformity, which may otherwise occur if only instrumented fixation is used.<br>- **Multi-Level Involvement**: For fractures that extend across multiple vertebral levels or that require stabilization over a larger area, arthrodesis can add long-term stability and prevent micro-motion between vertebrae, which could lead to hardware failure or chronic pain if left unfused.<br>- **Poor Bone Quality**: In patients with osteoporosis or poor bone quality, the addition of fusion may reduce the risk of hardware loosening, migration, or failure by ensuring a more stable construct.<br>**2. Benefits of Adding Arthrodesis to Instrumented Fixation**<br>- **Enhanced Long-Term Stability**: Arthrodesis creates a rigid fusion across the fracture site, which prevents any residual motion at the injured segment. This is particularly beneficial in unstable fractures where the risk of movement between vertebrae could lead to pain, deformity, or implant failure.<br>- **Prevention of Progressive Deformity**: By fusing the vertebrae, arthrodesis maintains proper spinal alignment and reduces the risk of progressive kyphotic deformity, which is common in burst fractures that are only instrumented. This is important for preserving posture, function, and long-term outcomes.<br>- **Reduced Risk of Hardware Complications**: Instrumented fixation alone may be susceptible to complications such as screw loosening or rod breakage over time, especially in cases of high mechanical stress. Fusion mitigates these risks by ensuring the vertebrae are biologically united, reducing strain on the hardware.<br>- **Improved Pain Control and Functional Outcomes**: Arthrodesis can lead to better pain control and improved functional outcomes in the long term, particularly in patients with unstable fractures. Stable fusion minimizes movement at the fracture site, which can help reduce pain and support earlier mobilization.<br>**3. Limitations and Considerations of Arthrodesis**<br>- **Loss of Motion at the Fused Segment**: Fusion inevitably limits mobility at the fused segment, which may reduce flexibility in the thoracic or lumbar spine. This can have functional implications, particularly in younger or more active patients.<br>- **Adjacent Segment Disease (ASD)**: Fusion transfers stress to adjacent segments, which may accelerate degeneration of these segments over time. This condition, known as adjacent segment disease, can lead to additional problems, including chronic pain and potential need for further surgery.

- **Increased Surgical Time and Risks**: Adding fusion to instrumented fixation increases the complexity of the surgery, extending operating time and potential blood loss. This can increase the risk of complications, including infection and delayed wound healing.
- **Higher Costs**: Fusion generally incurs higher costs due to additional materials (bone grafts or cages) and increased operating time.

4. Comparative Outcomes: Arthrodesis with Instrumented Fixation vs. Instrumented Fixation Alone

- **Stable vs. Unstable Fractures**: For relatively stable burst fractures without PLC injury or significant kyphosis, instrumented fixation alone is often sufficient, and the added benefits of fusion may be minimal. In these cases, fixation alone can stabilize the spine adequately while preserving more spinal mobility.
- **Better Outcomes in Unstable Cases with Fusion**: Studies suggest that in patients with unstable fractures, the addition of arthrodesis improves long-term outcomes by providing more robust stability and reducing the risk of complications associated with fixation alone, such as hardware failure or progressive deformity.
- **Patient-Reported Outcomes**: For unstable fractures requiring multi-level fixation, patients tend to report better outcomes in terms of pain relief and quality of life with the addition of arthrodesis. Fusion in these cases helps reduce micro-motion and irritation around the fracture site, which can contribute to chronic pain.

5. Guideline Recommendations and Clinical Practice

- **Selective Use of Arthrodesis**: Guidelines and clinical practice generally recommend the selective use of arthrodesis for patients with high-risk fracture characteristics, such as instability, significant kyphotic deformity, or compromised PLC integrity. In cases where fixation alone is adequate (stable fractures), fusion may be avoided to preserve mobility and reduce the risk of adjacent segment degeneration.
- **Patient-Specific Factors**: Decisions about adding fusion should consider the patient's age, activity level, and overall health. For younger patients or those with limited involvement of spinal stability, avoiding fusion may preserve spinal mobility without compromising outcomes.

Summary

Adding arthrodesis to instrumented fixation can improve clinical outcomes in patients with thoracic and lumbar burst fractures when:

- **There is significant instability** (e.g., PLC injury or multi-level involvement)

	• **Severe kyphotic deformity** is present or likely to develop<br>• **The patient has poor bone quality** that could affect hardware stability<br>For stable burst fractures or those with minimal kyphosis and no neurological compromise, instrumented fixation alone may be sufficient, avoiding the potential complications associated with fusion and preserving more spinal mobility.<br>**Conclusion**<br>Arthrodesis provides additional stability and prevents deformity progression in patients with unstable thoracic and lumbar burst fractures, often leading to improved pain relief, functional outcomes, and reduced hardware complications. However, for stable fractures, instrumented fixation alone may be adequate, offering a balanced approach that preserves mobility while ensuring sufficient stabilization. The decision should be tailored to the patient's fracture characteristics, stability, and overall health.
32.How does the use of minimally invasive techniques (including percutaneous instrumentation) affect outcomes in patients undergoing surgery for thoracic and lumbar fractures compared to conventional open techniques?	The use of minimally invasive techniques (MIT), including percutaneous instrumentation, has been shown to positively affect outcomes in patients undergoing surgery for thoracic and lumbar fractures compared to conventional open techniques. These techniques offer a less invasive approach to stabilization and fixation, aiming to reduce soft tissue damage while maintaining similar levels of stability and alignment. Here's an analysis of how minimally invasive techniques impact outcomes:<br>**1. Reduced Soft Tissue Damage and Muscle Preservation**<br>• **Minimized Muscle Disruption**: Minimally invasive techniques avoid the extensive muscle dissection required in open procedures, preserving more of the muscle's structure and function. This helps reduce postoperative pain, muscle weakness, and scarring, which are more common with open techniques.<br>• **Better Functional Recovery**: By sparing muscles, MIT can lead to improved postoperative muscle function and shorter recovery times, helping patients regain mobility and strength more quickly than with open surgery.<br>**2. Reduced Postoperative Pain**<br>• **Less Soft Tissue Trauma**: The smaller incisions and reduced muscle disruption in MIT are associated with less postoperative pain. Studies have shown that patients undergoing minimally invasive procedures report lower pain scores and often require less opioid analgesia than those treated with open surgery.<br>• **Improved Quality of Life**: Reduced pain contributes to improved quality of life, as patients can mobilize sooner and participate in physical therapy earlier, supporting better overall outcomes.<br>**3. Shorter Hospital Stay and Faster Recovery**

- **Early Mobilization**: With less pain and faster wound healing, patients treated with minimally invasive techniques are able to mobilize earlier, leading to shorter hospital stays and a quicker return to daily activities.
- **Reduced Complications from Prolonged Bed Rest**: Early mobilization reduces the risk of complications such as deep vein thrombosis (DVT), pulmonary complications, and muscle atrophy, which are more common with prolonged bed rest after open surgery.

4. Lower Risk of Infection and Wound Complications

- **Smaller Incisions**: The small incisions used in MIT reduce the risk of wound complications, such as infections, delayed healing, and dehiscence, which are more frequent with larger incisions in open surgery.
- **Reduced Blood Loss**: Minimally invasive techniques typically result in less intraoperative blood loss, which can lower the risk of transfusion-related complications and reduce overall surgical morbidity.

5. Comparable Stability and Alignment

- **Effectiveness of Percutaneous Instrumentation**: Percutaneous instrumentation provides similar biomechanical stability to open techniques, making it effective for stabilizing thoracic and lumbar fractures without compromising alignment or fusion rates.
- **Comparable Outcomes in Alignment and Fusion**: Studies show that minimally invasive techniques can achieve comparable outcomes in terms of spinal alignment, stability, and fusion rates for thoracic and lumbar fractures, especially when combined with modern imaging and navigation technologies for precise instrumentation placement.

6. Advantages for Specific Patient Populations

- **Older or High-Risk Patients**: MIT is especially beneficial for older adults or those with comorbidities who may be at higher risk of complications from open surgery. The reduced tissue trauma and quicker recovery associated with MIT make it safer and more manageable for these patients.
- **Patients with High Demand for Early Recovery**: Younger patients or those with active lifestyles benefit from minimally invasive approaches due to the quicker recovery, reduced pain, and minimal scarring, allowing them to return to daily activities and work sooner.

7. Limitations and Considerations of Minimally Invasive Techniques

- **Technical Complexity**: MIT procedures are technically demanding and may require specialized training and equipment. In

	complex fractures, MIT may be challenging to perform effectively, and the learning curve can impact outcomes.<br>• **Limited Direct Visualization**: Unlike open techniques, MIT relies heavily on intraoperative imaging (such as fluoroscopy or CT guidance) due to limited direct visualization, which can increase radiation exposure for both the patient and the surgical team.<br>• **Potential for Increased Operative Time**: While recovery time is shorter with MIT, operative time may be longer, particularly in cases with complex fractures or for surgeons early in their learning curve with these techniques.<br>**8. Comparative Outcomes: Minimally Invasive vs. Conventional Open Techniques**<br>• **Pain and Functional Recovery**: Patients treated with MIT generally experience less postoperative pain, quicker functional recovery, and improved quality of life compared to those undergoing open surgery.<br>• **Complication Rates**: MIT has been associated with lower rates of infection, wound complications, and blood loss. This reduces the likelihood of postoperative complications and supports faster recovery.<br>• **Equivalent Long-Term Outcomes**: Studies show that long-term outcomes for MIT are comparable to open techniques in terms of spinal alignment, stability, and fusion rates, indicating that minimally invasive methods do not compromise surgical success.<br>**Summary of Benefits of Minimally Invasive Techniques**<br>• **Reduced soft tissue disruption** and **muscle preservation** leading to quicker recovery and reduced postoperative pain<br>• **Lower risk of complications** such as infection, blood loss, and wound issues<br>• **Faster mobilization** and **shorter hospital stays**, which support early rehabilitation and functional recovery<br>• **Comparable outcomes** to open techniques in terms of stability, alignment, and fusion rates<br>**Conclusion**<br>Minimally invasive techniques, including percutaneous instrumentation, generally improve short-term outcomes in patients with thoracic and lumbar fractures by reducing postoperative pain, recovery time, and complication rates compared to conventional open techniques. For stable fractures, MIT achieves similar long-term outcomes as open surgery while offering the advantages of faster recovery and fewer complications. However, the technical complexity and need for specialized equipment are factors to consider, making MIT most beneficial when performed by experienced surgeons in appropriately selected patients.
33.Does the use of external bracing improve outcomes in the	The use of external bracing in the nonoperative treatment of neurologically intact patients with thoracic and lumbar burst fractures can

nonoperative treatment of neurologically intact patients with thoracic and lumbar burst fractures?	help improve outcomes by providing stability, reducing pain, and supporting the healing process. However, its effectiveness largely depends on the stability of the fracture and the specific characteristics of the injury. Here's an analysis of the role of external bracing in these patients and how it impacts clinical outcomes:<br>**1. Stabilization and Alignment Support**<br>● **Spinal Stability**: External bracing helps stabilize the spine in nonoperative cases by restricting movement at the fracture site. This can help prevent further displacement or misalignment of the fractured vertebrae during the healing process.<br>● **Control of Kyphotic Deformity**: Bracing provides support to prevent the progression of kyphotic deformity, which can develop in unstable fractures. By helping to maintain alignment, bracing reduces the risk of chronic postural issues that could arise from deformity.<br>**2. Pain Reduction and Improved Mobility**<br>● **Pain Relief**: Braces reduce the movement at the injury site, which often decreases pain and makes it easier for patients to engage in daily activities. This is particularly beneficial in the initial weeks following injury, as it helps manage acute pain.<br>● **Improved Functional Recovery**: By allowing patients to move with less pain, external bracing can help improve functional outcomes. Patients can mobilize sooner and may feel more confident moving around, which supports overall recovery.<br>**3. Enhanced Healing and Prevention of Further Injury**<br>● **Restricted Motion**: By limiting flexion, extension, and rotation, braces help to prevent micro-movements at the fracture site that could interfere with bone healing. This stability supports natural healing processes and reduces the risk of additional injury.<br>● **Guided Recovery**: Bracing can help patients adhere to activity limitations more effectively, reducing the risk of further injury due to accidental movement or excessive strain on the spine.<br>**4. Impact on Long-Term Outcomes**<br>● **Comparable Outcomes to Operative Treatment in Stable Fractures**: For neurologically intact patients with stable burst fractures, studies have shown that nonoperative treatment with bracing can achieve outcomes similar to those of surgical intervention in terms of pain, function, and return to activity.<br>● **Prevention of Progressive Deformity**: By preventing further collapse of the vertebral body, bracing can reduce the likelihood of progressive kyphotic deformity, which is associated with chronic pain and reduced functional capacity over the

long term.

5. Types of Braces and Their Effectiveness

- **Thoracolumbosacral Orthosis (TLSO)**: TLSO braces are commonly used for thoracic and lumbar fractures, as they provide rigid support that restricts flexion, extension, and rotation. TLSO braces are generally more effective than softer, flexible braces for burst fractures, as they provide more rigid stabilization.
- **Jewett and Hyperextension Braces**: These braces are designed to prevent flexion and support kyphotic correction, which can be beneficial in fractures with mild vertebral body collapse. They may be preferred in cases where kyphotic deformity is a concern.
- **Custom-Molded Braces**: In some cases, custom-molded braces can offer more precise support for individual patients, particularly when there are specific anatomical considerations or an unusual fracture pattern.

6. Limitations and Considerations of Bracing

- **Comfort and Compliance**: Bracing can be uncomfortable, especially when worn for long periods. Compliance is a key factor in the effectiveness of bracing, as noncompliance can limit the benefits of this treatment.
- **Limitations for Unstable Fractures**: Bracing is generally not sufficient for unstable burst fractures, where there is a risk of progressive deformity or neurological compromise. In these cases, surgery may be more appropriate to ensure adequate stabilization.
- **Duration of Use**: Prolonged bracing can lead to muscle weakness and atrophy due to limited movement. Patients may require physical therapy after brace removal to strengthen core muscles and restore spinal stability.

7. Comparative Studies and Evidence

- **Nonoperative Treatment in Neurologically Intact Patients**: Studies indicate that for neurologically intact patients with stable burst fractures, bracing combined with activity modification can produce outcomes similar to surgical intervention in terms of pain relief, functional recovery, and quality of life.
- **Effectiveness vs. No Brace**: Some studies suggest that for certain stable fractures, outcomes with bracing may not be significantly different from those achieved with careful activity modification alone, although bracing may still improve patient comfort and provide reassurance.

8. Guideline Recommendations

	- **Indication for Bracing in Stable Fractures**: Most guidelines recommend the use of bracing for stable thoracic and lumbar burst fractures in neurologically intact patients, as it supports stabilization and healing while avoiding the risks associated with surgery.<br>- **Avoidance of Bracing in Low-Risk Fractures**: For patients with very low-risk fractures and minimal collapse, some clinicians may opt for activity modification alone without bracing, depending on patient comfort and preferences.<br>**Summary**<br>The use of external bracing in the nonoperative treatment of neurologically intact patients with thoracic and lumbar burst fractures offers several benefits:<br>- **Stabilizes the fracture site** and prevents excessive movement, supporting bone healing<br>- **Reduces pain** by restricting motion at the injury site<br>- **Prevents progressive kyphotic deformity**, reducing the risk of long-term functional issues<br>- **Facilitates early mobility** by providing physical and psychological support<br>However, bracing may be less beneficial for very stable fractures or low-risk injuries, where activity modification alone could be sufficient.<br>**Conclusion**<br>For neurologically intact patients with stable thoracic and lumbar burst fractures, external bracing can improve clinical outcomes by providing stability, reducing pain, and supporting the healing process. Bracing is generally recommended for these patients as part of a nonoperative approach, particularly when there is a risk of progressive deformity or if pain is a concern. Bracing is most effective when patients are compliant and the duration of use is managed to avoid complications such as muscle weakness.
34.Which neurological assessment tools have demonstrated internal reliability and validity in the management of patients with thoracic and lumbar fractures (i.e., do these instruments provide consistent information between different care providers)?	Several neurological assessment tools have demonstrated internal reliability and validity for managing patients with thoracic and lumbar fractures, ensuring consistent information between different care providers. Reliable and valid tools are essential for accurately assessing neurological function, guiding treatment decisions, and monitoring recovery. Here are some of the most commonly used and validated assessment tools:<br>**1. American Spinal Injury Association (ASIA) Impairment Scale (AIS)**<br>- **Description**: The ASIA Impairment Scale, developed by the American Spinal Injury Association, is one of the most widely used tools for assessing neurological impairment in spinal cord injury (SCI) patients. It categorizes impairment from A

	(complete impairment) to E (normal function), based on sensory and motor function.<br>• **Components**: The ASIA exam assesses:<br>○ **Sensory Function**: Using a standardized pinprick and light touch sensation test across 28 dermatomes on each side.<br>○ **Motor Function**: Evaluating muscle strength in 10 key muscle groups on each side.<br>• **Reliability and Validity**:<br>○ **High Inter-Rater Reliability**: Studies demonstrate high inter-rater reliability, meaning different healthcare providers produce consistent results.<br>○ **Validated Tool**: The ASIA scale is validated for SCI assessment and provides consistent, comparable information, making it a gold standard for neurological assessment in thoracic and lumbar fractures with potential spinal cord involvement.<br>**2. Frankel Grading System**<br>• **Description**: The Frankel scale is a grading system that classifies spinal cord injuries into five categories, ranging from complete motor and sensory loss (Grade A) to normal function (Grade E). It is simpler than the ASIA scale and commonly used in trauma settings.<br>• **Reliability and Validity**:<br>○ **Good Inter-Rater Reliability**: The Frankel grading system has demonstrated good reliability and consistency among healthcare providers.<br>○ **Clinical Utility**: Although simpler than the ASIA scale, it is effective for quickly assessing neurological status and is reliable for monitoring changes over time in patients with thoracic and lumbar fractures.<br>**3. Motor Index Score (MIS)**<br>• **Description**: The Motor Index Score (MIS) is a quantitative measure based on the ASIA motor examination, assessing the strength of key muscles on each side of the body. The scores range from 0 (no movement) to 5 (normal strength), with a maximum score of 100.<br>• **Reliability and Validity**:<br>○ **High Inter-Rater Reliability**: The MIS has been shown to produce consistent scores between providers, making

	it a reliable tool for assessing motor impairment.<br>○ **Validity**: It provides a reliable, quantifiable measure of motor function, which is particularly useful for tracking changes in motor recovery over time in patients with spinal fractures.<br>**4. Spinal Cord Independence Measure (SCIM)**<br>• **Description**: The SCIM is a functional assessment tool designed specifically for patients with SCI. It measures the patient's ability to perform activities of daily living (ADLs) and assesses self-care, mobility, and respiratory function.<br>• **Reliability and Validity**:<br>○ **High Internal Consistency and Inter-Rater Reliability**: The SCIM has shown strong internal consistency and inter-rater reliability, making it a consistent measure for assessing functional independence across providers.<br>○ **Validated in SCI Populations**: Although primarily validated for SCI, it is useful for assessing functional outcomes in patients with thoracic and lumbar fractures, providing a broader view of functional recovery beyond sensory and motor function.<br>**5. Neurological Level of Injury (NLI)**<br>• **Description**: The NLI is determined as part of the ASIA assessment and identifies the most caudal level with intact sensory and motor function on both sides. It helps categorize the level and severity of the injury.<br>• **Reliability and Validity**:<br>○ **Good Reliability**: Studies have shown that the determination of NLI by trained providers is reliable and consistent across different assessors.<br>○ **Clinical Utility**: NLI is especially useful in the initial classification of injury severity and guiding the treatment approach, providing consistent information across different providers.<br>**6. Functional Independence Measure (FIM)**<br>• **Description**: The FIM assesses the patient's functional independence across several domains, including self-care, mobility, and communication. It is commonly used in rehabilitation settings.<br>• **Reliability and Validity**:<br>○ **High Reliability**: FIM has demonstrated high inter-rater and intra-rater reliability and is valid for assessing functional outcomes in patients with SCI.

- **Utility in Thoracic and Lumbar Fractures**: Although not specific to SCI, it provides useful data on functional abilities, particularly in assessing recovery and rehabilitation progress for patients with spinal fractures.

7. Visual Analog Scale (VAS) for Pain

- **Description**: The VAS is a simple, validated tool for assessing pain intensity. Patients mark their pain level on a 10-cm line, with 0 representing "no pain" and 10 representing "worst pain imaginable."
- **Reliability and Validity**:
  - **High Test-Retest Reliability**: The VAS provides consistent pain assessments across different providers, as it is based on patient self-report.
  - **Useful in Functional Assessment**: It is particularly valuable for tracking pain levels and evaluating the effectiveness of pain management strategies, which can be important for patients recovering from thoracic and lumbar fractures.

Summary of Neurological Assessment Tools

Tool	Primary Use	Reliability and Validity
**ASIA Impairment Scale**	Sensory and motor assessment in SCI	High inter-rater reliability and validity
**Frankel Grading System**	Quick grading of spinal cord injuries	Good inter-rater reliability
**Motor Index Score (MIS)**	Quantitative motor function assessment	High inter-rater reliability and validity
**SCIM**	Functional independence in SCI patients	High internal consistency and inter-rater reliability
**Neurological Level of Injury (NLI)**	Determining injury level	Good reliability
**FIM**	Functional independence in rehab	High reliability
**VAS for Pain**	Pain assessment	High test-retest reliability

Conclusion

The **ASIA Impairment Scale (AIS)**, **Frankel Grading System**, and **Motor Index Score (MIS)** are among the most reliable and validated tools for assessing neurological function in thoracic and lumbar fractures. The **SCIM** and **FIM** provide additional insight into functional independence, which is valuable for assessing outcomes and guiding rehabilitation. These tools ensure consistent assessments across

	different providers, enabling coordinated care and more accurate tracking of patient progress over time.
35.Are there any clinical findings (e.g., presenting neurological grade/function) in patients with thoracic and lumbar fractures that can assist in predicting clinical outcomes?	Yes, certain clinical findings in patients with thoracic and lumbar fractures can help predict clinical outcomes, including neurological recovery, functional independence, and long-term quality of life. Key predictors include the initial neurological status, the presence and severity of neurological deficits, spinal stability, and the degree of fracture displacement. Here's a breakdown of the main clinical findings that can assist in outcome prediction:<br>**1. Initial Neurological Grade or Function**<br>● **ASIA Impairment Scale (AIS) Grade**: Initial grading on the ASIA Impairment Scale (AIS) is one of the most significant predictors of clinical outcomes. Higher AIS grades (AIS A – complete injury) generally indicate a lower likelihood of neurological recovery, while lower grades (AIS D or E – partial or normal function) are associated with better recovery prospects.<br>● **Motor and Sensory Function**: Patients with preserved motor and sensory function at the time of injury (e.g., AIS D or E) have a better prognosis for functional recovery. Those with complete motor and sensory loss (AIS A) have a much lower chance of regaining significant function.<br>● **Frankel Scale**: Similarly, the Frankel grading system can predict outcomes, with higher Frankel grades indicating better neurological function and higher chances of regaining mobility and independence.<br>**2. Neurological Level of Injury (NLI)**<br>● **Location of Injury**: The level of spinal injury plays a critical role in predicting outcomes. Injuries in the thoracic region (particularly high thoracic levels) are more likely to result in significant loss of motor function and limitations in mobility, compared to injuries in the lumbar region, which may have a better functional prognosis.<br>● **Lower vs. Higher Thoracic Injuries**: Patients with lower thoracic or lumbar fractures typically retain more functional independence and have a higher likelihood of ambulation, while higher thoracic fractures (above T6) may impact autonomic function, including respiratory and cardiovascular control, potentially complicating recovery.<br>**3. Degree of Spinal Canal Compromise**<br>● **Percentage of Canal Compromise**: Radiological findings showing the percentage of spinal canal encroachment by retropulsed bone fragments are predictive of neurological outcomes. Greater than 50% spinal canal compromise is associated with poorer neurological recovery and a higher risk of residual deficits.

- **Presence of Cord Compression**: Evidence of spinal cord compression or neural element impingement, as seen on MRI or CT, is associated with worse neurological outcomes and is a predictor of lower functional recovery.

4. Spinal Stability and Posterior Ligamentous Complex (PLC) Integrity

- **Integrity of the PLC**: Injury to the posterior ligamentous complex (PLC), which stabilizes the spine, is a strong predictor of instability and poor outcomes. Patients with PLC disruption are more likely to experience progressive deformity, chronic pain, and functional limitations if not treated with surgery.
- **Stable vs. Unstable Fractures**: Stable fractures with intact PLC and minimal vertebral body collapse generally have better outcomes with nonoperative management, while unstable fractures are at higher risk for complications and poorer recovery if not stabilized surgically.

5. Presence of Kyphotic Deformity

- **Kyphotic Angle**: A significant kyphotic angle (>20 degrees) at the site of injury is associated with a poorer prognosis in terms of pain, mobility, and function. Progressive kyphosis can lead to chronic pain and postural issues, impacting quality of life.
- **Vertebral Height Loss**: Greater vertebral body height loss (typically more than 50%) is linked to a higher likelihood of kyphotic deformity, which can result in long-term complications if not managed appropriately.

6. Presence and Severity of Pain

- **Severe Pain at Presentation**: Severe pain, particularly if persistent and difficult to manage, can be an indicator of spinal instability or significant fracture displacement. Such patients may have poorer functional outcomes, as pain can limit mobility and delay recovery.
- **Impact on Functional Recovery**: Pain is also a predictor of the patient's ability to participate in rehabilitation, with higher pain levels often associated with slower functional recovery and lower quality of life.

7. Age and Comorbidities

- **Older Age**: Advanced age is associated with poorer outcomes due to reduced healing capacity, greater susceptibility to complications, and typically lower pre-injury functional status. Older patients with thoracic or lumbar fractures are less likely to achieve full functional recovery.
- **Presence of Comorbidities**: Comorbidities such as osteoporosis, cardiovascular disease, or diabetes can negatively impact

outcomes by slowing recovery, increasing complication risks, and limiting physical rehabilitation.

8. Timing and Type of Intervention

- **Early Surgical Intervention**: For patients with unstable fractures or neurological compromise, early surgical intervention (within 24–72 hours of injury) is associated with better neurological recovery, reduced hospital stays, and improved overall outcomes compared to delayed surgery.
- **Nonoperative Management in Stable Fractures**: Patients with stable fractures who can be managed nonoperatively often have good functional outcomes, provided they are compliant with bracing and activity restrictions. These patients tend to experience fewer complications and maintain higher levels of mobility and independence.

Summary of Key Predictive Clinical Findings

**Clinical Finding**	**Predictive Value for Outcomes**
**ASIA Impairment Scale (AIS) Grade**	Strong predictor of neurological and functional recovery; higher grades (AIS D, E) indicate better prognosis
**Neurological Level of Injury (NLI)**	Lower injury levels (lumbar) associated with better functional independence; high thoracic injuries (above T6) may impact autonomic function
**Spinal Canal Compromise**	>50% canal encroachment linked to poorer neurological recovery
**Posterior Ligamentous Complex (PLC) Integrity**	PLC disruption predicts instability, progressive deformity, and poorer recovery without surgical stabilization
**Kyphotic Deformity**	Significant kyphosis (>20 degrees) associated with chronic pain and postural issues
**Age and Comorbidities**	Older age and comorbidities predict slower recovery and lower functional outcomes
**Presence of Severe Pain**	Severe pain at presentation indicates instability or significant displacement; can limit rehabilitation and recovery

Conclusion

Clinical findings such as initial neurological grade, neurological level of injury, degree of spinal canal compromise, and spinal stability (including PLC integrity) are strong predictors of outcomes in patients with thoracic and lumbar fractures. These factors help guide

	treatment decisions and provide insight into the likely trajectory of recovery.
36.Does the active maintenance of arterial blood pressure after injury affect clinical outcomes in patients with thoracic and lumbar fractures?	Yes, the active maintenance of arterial blood pressure after injury can significantly affect clinical outcomes in patients with thoracic and lumbar fractures, particularly those with associated spinal cord injuries (SCI). Maintaining adequate blood pressure is critical for ensuring spinal cord perfusion, minimizing secondary injury, and promoting neurological recovery. Here's an analysis of how blood pressure management impacts outcomes and the clinical rationale behind it:<br>**1. Importance of Maintaining Spinal Cord Perfusion**<br>• **Preventing Secondary Injury**: After a spinal injury, there is often an immediate risk of secondary injury due to hypoperfusion, which can lead to spinal cord ischemia. Secondary injury worsens neurological outcomes by causing further damage to neurons and surrounding tissues.<br>• **Ensuring Adequate Oxygenation**: Maintaining blood pressure helps ensure that the spinal cord receives adequate oxygen and nutrients, which are essential for cell survival and repair processes. Hypotension can lead to tissue hypoxia, which impairs healing and increases the risk of poor neurological recovery.<br>**2. Guidelines for Blood Pressure Management in Spinal Cord Injury**<br>• **Target Mean Arterial Pressure (MAP)**: Clinical guidelines, including those from the American Association of Neurological Surgeons (AANS) and the Congress of Neurological Surgeons (CNS), recommend maintaining a target MAP of **85–90 mmHg** for the first 5–7 days post-injury in patients with spinal cord injury. This level of MAP helps optimize spinal cord perfusion and reduces the risk of ischemic injury.<br>• **Blood Pressure Goals for Lumbar Injuries**: While specific guidelines for thoracic and lumbar injuries without spinal cord involvement are less stringent, maintaining stable blood pressure is still important to support overall healing, especially in cases of severe trauma or in patients with other injuries.<br>**3. Clinical Evidence Supporting Blood Pressure Management**<br>• **Improved Neurological Outcomes**: Studies have shown that actively maintaining higher MAP levels in SCI patients is associated with improved neurological outcomes. Patients who achieve adequate spinal cord perfusion are more likely to experience partial recovery of motor and sensory function.<br>• **Reduced Risk of Complications**: Hypotension after injury is associated with a higher risk of complications, including increased rates of respiratory distress, infection, and delayed healing. By preventing hypotension, blood pressure

management reduces these risks, supporting a smoother recovery process.

- **Enhanced Functional Recovery**: Maintaining optimal blood pressure may improve functional recovery in patients with thoracic and lumbar fractures, as it helps prevent complications that could limit mobility and increase hospital stay durations.

4. Impact on Patients Without Spinal Cord Injury

- **Support for Healing and Stabilization**: In patients with thoracic and lumbar fractures who do not have SCI, active blood pressure maintenance is still beneficial as it supports tissue healing, reduces the risk of hypoperfusion-related complications, and ensures optimal recovery conditions.
- **Prevention of Hypotension-Related Morbidity**: Even without direct SCI, hypotension after trauma can lead to complications such as poor wound healing, pressure sores, and renal issues. Maintaining a stable blood pressure reduces the likelihood of these issues, which can indirectly impact recovery.

5. Management Strategies for Blood Pressure Control

- **IV Fluid Resuscitation**: Intravenous fluids are commonly used to maintain adequate blood volume and prevent hypotension, especially in the acute post-injury phase. This is essential for patients with trauma-induced blood loss or dehydration.
- **Vasoactive Medications**: For patients who cannot maintain adequate MAP with fluids alone, vasoactive medications such as norepinephrine or dopamine may be used to increase vascular tone and support blood pressure. These agents are particularly useful in SCI patients who may experience neurogenic shock, leading to severe hypotension.
- **Monitoring and Adjustments**: Continuous monitoring of blood pressure and MAP is necessary, especially in the first week post-injury. Adjustments to fluids and medications are often required to maintain consistent perfusion pressures.

6. Potential Risks and Limitations

- **Risk of Over-Resuscitation**: Excessive fluid administration can lead to fluid overload, pulmonary edema, and cardiac complications, particularly in older patients or those with pre-existing heart conditions.
- **Risk of Hypertension-Related Complications**: Although high MAP is beneficial for spinal cord perfusion, overly aggressive use of vasoactive medications can cause hypertension, which may lead to complications such as intracranial pressure elevation or organ damage.

7. Other Factors Affecting Outcomes

- **Blood Pressure in the Context of Other Injuries**: In patients with multiple traumas, managing blood pressure can be more

challenging. Prioritizing MAP maintenance for spinal cord perfusion must be balanced with managing other injuries, such as traumatic brain injury, where elevated blood pressure could pose a risk.

- **Individual Patient Considerations**: Age, comorbidities, and baseline blood pressure affect how patients respond to MAP management. Personalized approaches to blood pressure management are often needed to avoid complications while achieving optimal perfusion.

Summary of Blood Pressure Management's Impact on Clinical Outcomes

Outcome	Impact of Active BP Maintenance
**Neurological Recovery**	Higher MAP supports spinal cord perfusion, reducing ischemia and secondary injury, which can improve motor and sensory recovery
**Reduction in Complications**	Prevents hypotension-related complications, such as hypoxia, infection, and delayed healing, which supports smoother recovery
**Functional Recovery**	Improved blood flow and fewer complications contribute to better functional outcomes and shorter hospital stays
**Tissue Healing**	Consistent perfusion supports bone and tissue healing, reducing morbidity and supporting structural recovery of the spine

Conclusion

Active maintenance of arterial blood pressure after thoracic and lumbar fractures, especially in cases with spinal cord injury, positively affects clinical outcomes by supporting spinal cord perfusion, minimizing secondary injury, and enhancing neurological and functional recovery. For SCI patients, maintaining a MAP of 85–90 mmHg for the first 5–7 days post-injury is recommended to optimize outcomes. Even in patients without SCI, stable blood pressure supports healing and reduces complications, indirectly promoting better recovery. This approach requires careful management and continuous monitoring to balance the benefits of adequate perfusion with the risks of fluid overload and hypertension-related complications.

37.Are there classification systems for fractures of the thoracolumbar spine that have been shown to be internally valid and reliable (i.e., do these instruments provide consistent	Yes, several classification systems for thoracolumbar spine fractures have demonstrated internal validity and reliability, providing consistent information across different care providers. These classification systems are designed to standardize the assessment of thoracolumbar fractures, guide treatment decisions, and predict outcomes. Here are some of the most commonly used and validated

information between different care providers)?	classification systems for thoracolumbar fractures:<br>**1. Thoracolumbar Injury Classification and Severity Score (TLICS)**<br>● **Description**: Developed by the Spine Trauma Study Group, the TLICS system classifies thoracolumbar injuries based on three key components:<br>○ **Injury Morphology**: Compression (1 point), burst (2 points), translation/rotation (3 points), or distraction (4 points)<br>○ **Posterior Ligamentous Complex (PLC) Integrity**: Intact (0 points), indeterminate (2 points), disrupted (3 points)<br>○ **Neurological Status**: Intact (0 points), nerve root injury (2 points), complete cord injury (2 points), incomplete cord injury (3 points)<br>● **Scoring and Treatment Guidance**: Scores of 4 or higher generally indicate that surgery is recommended, while scores below 4 suggest nonoperative management.<br>● **Reliability and Validity**:<br>○ **High Inter-Rater Reliability**: TLICS has been shown to have good inter-rater reliability, with different providers achieving consistent scores across cases.<br>○ **Predictive Validity**: The TLICS score correlates well with treatment recommendations, making it a reliable guide for surgical versus nonoperative decisions. Studies support its predictive value for outcomes based on the severity of injury.<br>**2. AO Spine Thoracolumbar Injury Classification System**<br>● **Description**: The AO Spine classification system, developed by the AO Spine Injury Group, is based on injury morphology, neurological status, and the patient's specific modifiers (e.g., comorbidities). It categorizes fractures into three primary types:<br>○ **Type A**: Compression injuries with minimal structural instability<br>○ **Type B**: Distraction injuries, involving failure of tension band structures<br>○ **Type C**: Translational injuries, which are highly unstable with displacement in multiple directions<br>● **Subcategories**: Each type has further subcategories based on the injury's severity and morphology.<br>● **Reliability and Validity**:<br>○ **Good Inter-Rater Reliability**: The AO Spine classification system has shown good inter-rater reliability, making it consistent across providers.

    - **Validity and Clinical Utility**: Studies validate this system as a reliable predictor of treatment recommendations and outcomes, and it is commonly used worldwide for its comprehensive structure and guidance on instability and neurological risk.

3. Denis Classification System (Three-Column Model)

- **Description**: The Denis system classifies fractures based on the concept of three anatomical columns in the spine:
    - **Anterior Column**: Anterior half of the vertebral body, disc, and anterior longitudinal ligament
    - **Middle Column**: Posterior half of the vertebral body, disc, and posterior longitudinal ligament
    - **Posterior Column**: Posterior elements, including pedicles, facet joints, and ligamentous structures
- **Fracture Types**: Denis describes fractures as compression, burst, seat-belt (Chance), or fracture-dislocation, depending on which columns are involved.
- **Reliability and Validity**:
    - **Moderate Inter-Rater Reliability**: The Denis classification has moderate reliability among providers, especially in identifying which columns are involved in the fracture.
    - **Validity in Predicting Stability**: The three-column model has good clinical validity for assessing spinal stability and has been useful for guiding treatment decisions, though it is less detailed than more recent systems.

4. McAfee Classification System

- **Description**: The McAfee system, an expansion of the Denis model, classifies thoracolumbar fractures into six types based on their morphology and instability:
    - **Wedge-Compression**
    - **Stable Burst**
    - **Unstable Burst**
    - **Chance**
    - **Translational**
    - **Distraction**
- **Reliability and Validity**:
    - **Moderate Inter-Rater Reliability**: Studies have found moderate reliability in classifying fractures with this system,

although it remains less commonly used today.

  - **Clinical Usefulness**: It provides useful information on instability, but due to moderate reliability, it is often supplemented with other classification systems.

5. Load-Sharing Classification (LSC)

- **Description**: The Load-Sharing Classification assesses thoracolumbar burst fractures based on:
  - **Degree of Vertebral Body Comminution** (fracture fragmentation)
  - **Degree of Fracture Displacement**
  - **Degree of Posterior Element Involvement**
- **Scoring**: Each factor is scored from 1 to 3, with higher scores suggesting greater instability and a higher likelihood of requiring surgical stabilization.
- **Reliability and Validity**:
  - **Good Inter-Rater Reliability**: LSC has demonstrated reliable scoring between different providers.
  - **Predictive Validity for Surgical Decision-Making**: The LSC score is validated for guiding surgical decision-making, especially in predicting the need for anterior column reconstruction.

6. Gertzbein and Ratliff Classification System

- **Description**: This system classifies thoracolumbar fractures based on radiographic analysis and the presence of neurological deficits. Fractures are grouped into stable and unstable types, focusing on structural integrity and the potential for neurological compromise.
- **Reliability and Validity**:
  - **Moderate Inter-Rater Reliability**: It has been found to have moderate reliability but is less commonly used than TLICS or AO Spine.
  - **Limited Use in Modern Practice**: While valid for assessing stability, it is primarily used in research contexts and has been largely replaced by newer systems.

Comparison of Classification Systems

Classification System	Key Components	Reliability	Validity/Clinical Utility
**TLICS**	Morphology, PLC integrity, neurological	High inter-rater	Good predictive validity for treatment

	status	reliability	decisions
**AO Spine**	Morphology, neurological status, modifiers	Good inter-rater reliability	Validated globally for guiding treatment and predicting outcomes
**Denis Three-Column**	Anterior, middle, and posterior columns	Moderate reliability	Useful for stability assessment
**McAfee**	Six fracture types based on morphology	Moderate reliability	Useful but less commonly used today
**Load-Sharing Classification (LSC)**	Comminution, displacement, posterior element involvement	Good reliability	Predicts need for anterior column reconstruction
**Gertzbein and Ratliff**	Stability and neurological status	Moderate reliability	Valid but less frequently used

Summary

- **TLICS and AO Spine** classifications are the most widely used, with high reliability and clinical utility, making them effective tools for guiding treatment and predicting outcomes.
- **Denis Three-Column Model** is useful for understanding stability but less detailed than TLICS or AO Spine.
- **Load-Sharing Classification (LSC)** is reliable for predicting the need for surgical stabilization, especially anterior reconstruction in burst fractures.

Conclusion

The **TLICS** and **AO Spine** classification systems are the most reliable and valid for thoracolumbar fractures, with high inter-rater reliability and strong clinical utility in guiding treatment and predicting outcomes. The **Denis Three-Column Model** and **Load-Sharing Classification** are also valuable tools, particularly for assessing stability and surgical needs. These systems provide consistent, standardized information across providers, facilitating effective communication, treatment planning, and outcome prediction.

38.In treating patients with thoracolumbar fractures, does employing a formally tested classification system for treatment decision-making affect clinical outcomes?

Yes, employing a formally tested classification system for treatment decision-making in patients with thoracolumbar fractures can positively affect clinical outcomes. Using a structured and validated classification system, such as the **Thoracolumbar Injury Classification and Severity Score (TLICS)** or the **AO Spine Thoracolumbar Injury Classification**, provides several benefits that contribute to more consistent and effective patient care. Here's how these systems impact clinical outcomes:

1. Improved Consistency in Treatment Decisions

- **Standardized Assessment**: Classification systems provide a standardized approach to assessing fracture characteristics (e.g.,

stability, morphology, neurological involvement), allowing for consistent evaluation across different care providers. This consistency helps reduce variability in treatment decisions, leading to more predictable and uniform care.

- **Clear Treatment Guidelines**: Systems like TLICS and AO Spine offer clear, evidence-based guidance on when to opt for surgical versus nonoperative treatment, helping clinicians make informed and consistent treatment choices. For example, TLICS scores of 4 or higher typically suggest the need for surgery, while lower scores indicate suitability for conservative management.

2. Improved Alignment with Evidence-Based Practices

- **Guided Decision-Making**: Classification systems integrate key factors (e.g., fracture morphology, posterior ligamentous complex integrity, neurological status) that are predictive of outcomes. This ensures that treatment decisions align with best practices, leading to more favorable results for patients.
- **Reduction in Overtreatment and Undertreatment**: By clearly identifying patients who would benefit most from surgical intervention (e.g., unstable fractures) and those who can be managed nonoperatively (e.g., stable fractures without neurological deficits), classification systems help avoid unnecessary surgery and reduce the risk of undertreating high-risk fractures.

3. Enhanced Prediction of Stability and Outcomes

- **Identification of Unstable Fractures**: Systems like TLICS and the Denis Three-Column Model assess spinal stability, a critical factor in determining treatment. Accurately identifying unstable fractures allows for timely stabilization, which prevents further injury, progressive deformity, and chronic pain—factors that negatively impact clinical outcomes.
- **Prognostic Value**: The predictive components within these systems (such as TLICS scoring) are associated with outcomes like neurological recovery, deformity risk, and functional improvement. This allows clinicians to make better prognostic judgments and set appropriate expectations for patients and their families.

4. Enhanced Communication Among Providers

- **Consistency in Multidisciplinary Settings**: Classification systems provide a common language and framework, facilitating communication among multidisciplinary teams, including spine surgeons, trauma specialists, and radiologists. This enhances collaboration, particularly in complex cases where decisions on surgery, bracing, and rehabilitation require input from multiple specialties.

- **Improved Continuity of Care**: Standardized classification ensures continuity of care as patients transition between providers (e.g., from emergency care to rehabilitation), reducing miscommunication and errors in treatment planning.

5. Reduction in Complications and Improved Safety

- **Guided Surgical Indications**: Classification systems improve decision-making by helping identify cases that truly require surgery versus those that can heal with conservative management. This reduces the risk of surgical complications, such as infection, hardware failure, and adjacent segment disease, by avoiding unnecessary operations.
- **Fewer Revisions**: By accurately determining the need for surgery and type of stabilization, classification systems help reduce the incidence of failed conservative treatments or inadequate fixation, which often lead to revision surgeries. Properly classifying and treating the fracture type from the outset contributes to better long-term outcomes and reduces morbidity.

6. Tailored Rehabilitation and Recovery Planning

- **Customizing Post-Treatment Care**: By determining stability and potential for neurological recovery, classification systems help guide post-treatment rehabilitation. For example, patients with stable fractures may benefit more from early mobilization, while those with unstable fractures may require prolonged bracing or cautious mobilization.
- **Setting Realistic Expectations**: Systems that assess neurological status and stability allow clinicians to set realistic recovery expectations for patients, enabling better preparation for the rehabilitation process and potential limitations.

Evidence of Improved Clinical Outcomes with Classification Systems

- **TLICS and AO Spine Classification Studies**: Research has shown that these systems not only standardize treatment decisions but also correlate well with outcomes. For example, patients treated according to TLICS scoring have shown improved alignment with treatment guidelines, which translates to better functional and neurological outcomes.
- **Reduced Rates of Mismanagement**: Studies indicate that using classification systems can reduce rates of mismanagement, such as the inappropriate choice of nonoperative treatment in unstable fractures, which can lead to progressive deformity or delayed neurological deterioration.

Summary of Benefits of Using Classification Systems

**Benefit**	**Impact on Clinical Outcomes**
**Consistency in Treatment**	Reduces variability, leading to uniform care and predictable results

**Evidence-Based Decisions**	Aligns with best practices, avoiding unnecessary or insufficient interventions
**Enhanced Prognostic Accuracy**	Assists in identifying fracture stability and predicting recovery potential
**Improved Communication**	Standardizes language among providers, enhancing multidisciplinary care
**Reduction in Complications**	Minimizes overtreatment and undertreatment, lowering complication rates
**Guided Rehabilitation**	Tailors post-treatment care to fracture stability and neurological status

Conclusion

Employing formally tested classification systems like TLICS and AO Spine in the treatment of thoracolumbar fractures leads to better clinical outcomes by providing consistent, evidence-based guidance for treatment decisions. These systems improve patient safety, predict outcomes more accurately, and enhance communication among care providers, ultimately leading to more effective and coordinated care.
